# Advancing Nanogenerators: The Role of 3D-Printed Nanocomposites in Energy Harvesting

**DOI:** 10.3390/polym17101367

**Published:** 2025-05-16

**Authors:** Riyamol Kallikkoden Razack, Kishor Kumar Sadasivuni

**Affiliations:** Center for Advanced Materials, Qatar University, Doha P.O. Box 2713, Qatar; riyakrmol@gmail.com

**Keywords:** 3D printing, nanogenerators, nanocomposites, 3D-printed nanogenerators, flexible energy harvesting

## Abstract

Nanogenerators have garnered significant scholarly interest as a groundbreaking approach to energy harvesting, encompassing applications in self-sustaining electronics, biomedical devices, and environmental monitoring. The rise of additive manufacturing has fundamentally transformed the production processes of nanocomposites, allowing for the detailed design and refinement of materials aimed at optimizing energy generation. This review presents a comprehensive analysis of 3D-printed nanocomposites in the context of nanogenerator applications. By employing layer-by-layer deposition, multi-material integration, and custom microstructural architectures, 3D-printed nanocomposites exhibit improved mechanical properties, superior energy conversion efficiency, and increased structural complexity when compared to their conventionally manufactured counterparts. Polymers, particularly those with inherent dielectric, piezoelectric, or triboelectric characteristics, serve as critical functional matrices in these composites, offering mechanical flexibility, processability, and compatibility with diverse nanoparticles. In particular, the careful regulation of the nanoparticle distribution in 3D printing significantly enhances piezoelectric and triboelectric functionalities, resulting in a higher energy output and greater consistency. Recent investigations into three-dimensional-printed nanogenerators reveal extraordinary outputs, encompassing peak voltages of as much as 120 V for BaTiO_3_-PVDF composites, energy densities surpassing 3.5 mJ/cm^2^, and effective d_33_ values attaining 35 pC/N, thereby emphasizing the transformative influence of additive manufacturing on the performance of energy harvesting. Furthermore, the scalability and cost-effectiveness inherent in additive manufacturing provide substantial benefits by reducing material waste and streamlining multi-phase processing. Nonetheless, despite these advantages, challenges such as environmental resilience, long-term durability, and the fine-tuning of printing parameters remain critical hurdles for widespread adoption. This assessment highlights the transformative potential of 3D printing in advancing nanogenerator technology and offers valuable insights into future research directions for developing high-efficiency, sustainable, and scalable energy-harvesting systems.

## 1. Introduction

The escalating demand for sustainable and adaptable energy sources has catalyzed comprehensive investigations into pioneering energy-harvesting technologies. In light of the burgeoning presence of wearable electronics, biomedical implants, and Internet of Things [IoT] devices, the necessity for autonomous power sources has intensified significantly [1]. Usual energy storage systems, comprising lithium-ion batteries, suffer from shortcomings such as limited durability, ecological issues, and the demand for constant recharging, thus proving inadequate for next-generation uses [2,3]. Consequently, researchers have delved into alternative energy-harvesting techniques capable of generating electricity from ambient sources, encompassing mechanical motion, thermal gradients, and electromagnetic waves [4,5]. Nanogenerators have emerged as a viable solution to these issues, providing self-sustaining and environmentally friendly energy-harvesting capabilities [6]. They function by converting minute-scale environmental energy into electrical energy, facilitating applications in self-powered sensors, intelligent textiles, and implantable medical devices [7,8]. Nonetheless, traditional manufacturing methodologies impose considerable constraints on the scalability, longevity, and efficacy of nanogenerators, thereby necessitating innovative fabrication techniques to enhance their performance [9]. This has prompted a burgeoning interest in the integration of advanced nanocomposite materials with three-dimensional printing to fabricate next-generation nanogenerators [10].

Nanogenerators can be classified based on how they convert energy into types such as triboelectric nanogenerators [TENGs], piezoelectric nanogenerators [PENGs], and pyroelectric nanogenerators [11]. TENGs facilitate charge transfer between substances with varying electron affinities, transforming mechanical motion into electrical energy [11,12]. These devices have been effectively employed in extracting energy from human motion, wind energy, and even vibrational energy from infrastructures [13]. PENGs exploit the piezoelectric effect, producing electrical energy when exposed to mechanical stress, which renders them as particularly advantageous for applications in biomedical implants, structural health monitoring, and flexible electronic devices [14,15]. Pyroelectric nanogenerators, which utilize fluctuations in temperature for energy conversion, have also attracted considerable interest for their potential in thermal energy-harvesting applications [16]. Hybrid nanogenerators, which amalgamate various energy-harvesting mechanisms, have exhibited an improved efficiency and adaptability across a spectrum of applications [17,18]. These technological advancements have facilitated the creation of self-sustaining gas sensors, medical implants, and intelligent textiles that are capable of real-time health monitoring [19,20]. Nevertheless, the pursuit of scalable manufacturing while preserving a high mechanical robustness and energy conversion efficiency continues to pose a significant challenge [11,21].

The integration of three-dimensional [3D] printing technology with nanocomposite materials introduces a revolutionary methodology to overcome the limitations inherent in conventional nanogenerator fabrication techniques. Three-dimensional printing serves as a means to achieve precise adjustments in structural architecture, enhances material utilization, and allows for the incorporation of multifunctional nanomaterials, thus improving energy conversion efficiency [22,23]. By leveraging its potential to create intricate structures, 3D printing supports the advancement of nimble, lightweight, and optimized nanogenerators that are finely tuned for specific purposes [24,25]. Nanocomposites that merge substances such as carbon nanotubes, metal oxides, and piezoelectric polymers have significantly enhanced the electrical and mechanical properties of nanogenerators fabricated through 3D printing [26,27]. Recent advancements in multi-material additive manufacturing now enable the precise integration of conductive, dielectric, and piezoelectric components within a single printed framework, thereby maximizing the efficiency of energy harvesting [28,29]. These innovations have also paved the way for the development of self-healing nanogenerators and resilient, flexible energy storage solutions, thereby enhancing the longevity and performance of devices across diverse environmental conditions [30,31].

3D-printed nanogenerators have shown significant promise across various fields, including biomedical applications, wearable electronics, and environmental sensing. Within biomedical devices, these self-sustaining nanogenerators improve real-time health monitoring, drug delivery, and remote patient diagnostics [20,32]. Wearable triboelectric nanogenerators [TENGs] and piezoelectric nanogenerators [PENGs] have been effectively integrated into smart fabrics for energy harvesting driven by movement and for tracking biological signals [15,33]. Furthermore, self-powered microsystems designed for fall detection, ultraviolet sensing, and marine corrosion prevention highlight their importance in healthcare and environmental protection [19,21]. The incorporation of 3D printing in energy-harvesting technologies has also promoted the development of sustainable materials and environmentally friendly manufacturing methods. For instance, additive manufacturing techniques allow for the recycling of plastic waste into functional nanogenerators, thereby reducing material waste and supporting the principles of a circular economy [34]. Moreover, the design flexibility offered by 3D printing facilitates the creation of hybrid fillers and hierarchical nanostructures, significantly boosting the energy conversion efficiency of nanogenerators [23,35].

Despite the plethora of advancements in the domain of 3D-printed nanogenerators, several challenges remain unresolved. The mechanical robustness and long-term reliability of 3D-printed constructs continue to be significant areas of apprehension, as persistent mechanical stress and environmental variables have the potential to compromise device efficacy [11,25]. Furthermore, the optimization of the interfacial adhesion between nanocomposite materials and printed matrices is imperative for realizing elevated energy conversion efficiencies [14,28]. Subsequent research endeavors must prioritize the enhancement of the reliability of 3D-printed nanogenerators through innovative material formulations and sophisticated computational modeling techniques [23,29]. The incorporation of machine learning and artificial intelligence methodologies within material design can further expedite the optimization of nanogenerator architectures for specific applications [36]. In addition, large-scale manufacturing methodologies, such as roll-to-roll printing and hybrid additive manufacturing, exhibit considerable potential for the commercialization of flexible energy-harvesting systems [33,35]. The convergence of nanogenerator technology and additive manufacturing is anticipated to unveil new avenues in sustainable energy solutions. By harnessing advanced material science and pioneering fabrication methodologies, researchers may facilitate the development of next-generation nanogenerators characterized by improved performance, adaptability, and endurance [17,22]. As these technologies progress, their incorporation into biomedical devices, intelligent infrastructure, and autonomous IoT systems will significantly broaden their influence in practical applications [20,31].

This review analyzes advancements in 3D-printed nanocomposites for energy harvesting, focusing on enhancing nanogenerator performance and applications. The synergy of nanogenerators and 3D printing has facilitated the creation of flexible, efficient, and scalable energy-harvesting systems suitable for wearable electronics, biomedical devices, and IoT infrastructures [11]. A comprehensive understanding of nanogenerator principles and mechanisms is vital for optimizing their design and functionality. Key principles, including triboelectric, piezoelectric, and hybrid techniques, have been investigated to improve efficiency and adaptability [8]. The optimization of energy-harvesting systems through 3D printing has garnered considerable interest recently. Additive manufacturing provides precise control, efficient material use, and the seamless integration of nanocomposite materials, enhancing the energy conversion efficiency [35]. Advancements in nanocomposite materials have facilitated the development of autonomous devices, wherein the incorporation of conductive, piezoelectric, and dielectric elements has markedly augmented the output of nanogenerators [29]. However, obstacles hinder the extensive implementation of 3D-printed nanogenerators. Mechanical durability, output stability, and scalability pose significant concerns, necessitating a further exploration of material formulations, structural designs, and manufacturing techniques. By overcoming these obstacles, researchers can enhance nanogenerator designs for improved efficiency and reliability [17]. This review aims to present a thorough overview of developments, identify major challenges, and suggest future research directions to promote the widespread implementation of 3D-printed nanogenerators.

3D-printed nanocomposite-based nanogenerators are crucial for advancing sustainable energy solutions. Their diverse applications highlight their importance across multiple disciplines, supporting various emerging technologies [32]. These devices efficiently harvest ambient energy from various sources, positioning them for innovative uses in smart healthcare, autonomous sensors, and renewable energy [20]. Ongoing progress in material science and fabrication methods enhances the efficiency and durability of nanogenerators. Future studies must prioritize optimizing material properties and structural designs to improve energy conversion efficiency [31]. The addition of self-healing capabilities could prolong the lifespan of devices by facilitating real-time repairs [37]. Furthermore, hybrid systems that combine multiple energy-harvesting methods could optimize the power output and performance [36]. A vital aspect is improving large-scale manufacturing processes for the commercial viability of 3D-printed nanogenerators. Despite advancements in additive manufacturing, scalable and cost-effective production remains a significant hurdle. Innovative methods like roll-to-roll printing and multi-material additive manufacturing are essential for mass production [23]. Overcoming these challenges will enable the realization of flexible energy solutions, fostering sustainable and self-powered technologies [17].

## 2. Fundamentals of Nanogenerators for Flexible Energy Harvesting

Nanogenerators are paving the way for groundbreaking advancements in energy collection, serving as self-sufficient power sources for wearable tech, healthcare innovations, and IoT frameworks. These gadgets transform mechanical, thermal, and diverse environmental energies into electrical energy using nanoscale materials and frameworks. Their inherent flexibility and adaptability make them an exemplary selection for integrating energy harvesting with advanced functional materials, ensuring incessant and efficient power generation for next-generation technological applications [38].

Nanogenerators are categorized into triboelectric nanogenerators [TENGs], piezoelectric nanogenerators [PENGs], and hybrid nanogenerators, predicated on distinct energy conversion mechanisms. TENGs function based on the triboelectric effect, wherein charge transfer transpires due to the contact electrification between disparate materials, resulting in the generation of an electric potential difference that facilitates the flow of current [39]. Conversely, PENGs employ piezoelectric materials that produce electricity in response to mechanical deformation, rendering them apt for harvesting biomechanical energy from human movement and ambient vibrations [40]. Hybrid nanogenerators synthesize various energy conversion principles like piezoelectric, triboelectric, and pyroelectric effects to refine the power generation efficiency and flexibility regarding numerous energy sources [41].

### 2.1. Triboelectric Nanogenerators [TENGs]

**Figure 1:**
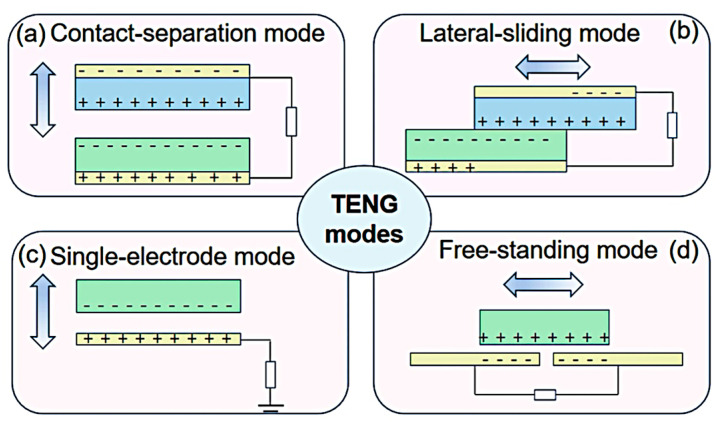
Different operational modes of TENGs [43].

Triboelectric nanogenerators [TENGs] rely on the interplay between triboelectrification and electrostatic induction, enabling the conversion of mechanical energy into electrical energy. This phenomenon occurs when two distinct materials with different electron affinities come into contact and then separate, leading to the transfer of charge, which creates a potential difference that drives the electron flow through an external circuit [39]. This principle has been effectively utilized in various applications, including self-sustaining sensors, environmental monitoring, and large-scale energy harvesting [42]. The effectiveness of energy conversion in TENGs depends on their operational mode, which influences the mechanisms of charge transfer and the characteristics of the electrical output. There are four primary operational modes, as shown in Figure 1:

Vertical Contact–Separation Mode: In the vertical contact–separation mode, two triboelectric materials are induced into contact through an external force and subsequently separated, resulting in charge transfer between the two [44]. A potential difference is established upon the separation of the materials, which facilitates electron flow between the electrodes to equilibrate the charge distribution [45]. The output performance of this mode is significantly influenced by the intrinsic properties of the materials, the area of contact, and the magnitude of the applied pressure. This mode finds extensive applications in self-sufficient electronic devices, biomechanical energy harvesting, and pressure-sensitive sensors [46]. Recent innovations have investigated soft polymeric materials and nanostructured surfaces to augment charge transfer efficiency [47]. Lateral Sliding Mode: The lateral sliding mode functions when two triboelectric layers engage in relative motion in a parallel manner, inducing charge separation and subsequent electron flow between the electrodes [48]. This mode is particularly advantageous for capturing energy derived from shear forces, such as those produced by human movement, wind, and water flow [49]. The sliding motion perpetually generates and redistributes charges, thereby providing a consistent energy output suitable for powering diminutive electronic devices [49]. Innovative designs incorporating micro- and nanoscale surface textures have markedly enhanced the charge transfer efficiency and longevity of TENGs functioning in this mode [50]. Single-Electrode Mode: The single-electrode mode permits energy generation without necessitating both triboelectric layers to be directly interfaced with electrodes. In this configuration, one triboelectric material serves as the active surface, while the external environment [e.g., the human body] interacts with it to facilitate charge transfer [51]. This mode is exceptionally advantageous for wearable electronics, touch-sensitive interfaces, and biomedical applications, wherein one electrode remains grounded while charge transfer transpires via air breakdown or capacitive coupling [52]. Single-electrode TENGs have been adeptly integrated into self-powered health monitoring systems, including motion-tracking wearables and implantable sensors [53]. Freestanding Mode: In the freestanding configuration, a mobile triboelectric layer functions between two stationary electrodes, facilitating dynamic charge transfer via electrostatic induction [54]. This configuration proves particularly efficacious for extensive energy-harvesting applications, such as the conversion of ocean wave energy and the harvesting of wind energy, wherein a freely moving object perpetually engages with the electrodes to generate electrical power [41]. Recent investigations have introduced non-contact TENG designs aimed at minimizing wear and enhancing long-term operational stability [55]. These advancements have culminated in the creation of highly efficient, low-maintenance energy harvesters suitable for both industrial and environmental applications [56].

The ongoing advancement of TENGs has focused on optimizing material selection, structural design, and hybrid energy-harvesting methodologies. The incorporation of sophisticated triboelectric materials, including high-performance polymers and two-dimensional nanomaterials, has significantly enhanced charge generation capabilities [57]. Furthermore, hybrid TENGs that integrate piezoelectric, pyroelectric, and photovoltaic effects have a notably improved overall energy output and efficiency [58]. Future research efforts are expected to explore biodegradable TENGs for sustainable energy solutions, along with the development of AI-integrated self-powered sensors for a real-time data analysis relevant to wearable technology and IoT systems [59].

### 2.2. Piezoelectric Nanogenerators [PENGs]

Piezoelectric nanogenerators [PENGs] represent a pivotal technological advancement for transmuting mechanical energy into electrical energy through the utilization of the piezoelectric effect. This phenomenon manifests in specific crystalline substances that produce an electrical charge upon the application of mechanical stress, which may encompass actions such as bending, stretching, or compression [59]. Following their inception, PENGs have attracted considerable scholarly interest in the realm of energy-harvesting applications, especially concerning wearable electronics, biomedical instruments, and structural health monitoring systems [40]. Their proficiency in effectively generating power from biomechanical movements and environmental vibrations renders them exceptionally apt for self-sustaining electronic systems [45].

**Figure 2:**
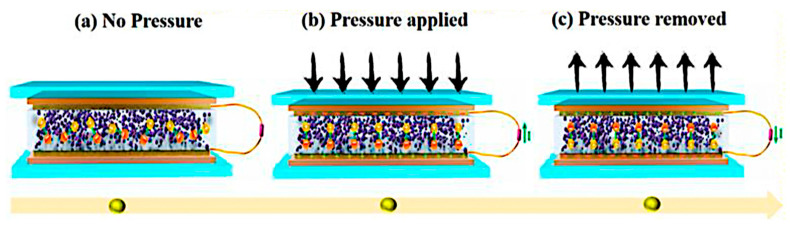
Working mechanism of Piezoelectric Nanogenerator [66].

The essential operational principle underlying PENGs is predicated on the direct piezoelectric effect, wherein an external mechanical force engenders an electrical potential through the displacement of charge carriers embedded within the piezoelectric material. Upon the application of a mechanical stimulus, such as compression or bending, the internal dipole moments within the material undergo realignment, culminating in charge separation and an induced voltage across the electrodes [60]. This electrical energy can subsequently be either stored or directly harnessed to power diminutive electronic devices [61]. This basic principle of piezoelectric nanogenerators is shown in Figure 2. Piezoelectric nanogenerators conventionally function in two predominant configurations contingent upon the mechanical force applied. In the vertical compression mode, the force is exerted perpendicularly to the material’s surface, resulting in direct charge displacement. This configuration is extensively employed in scenarios necessitating pressure-based energy harvesting, such as energy collection from footfalls in smart flooring systems or pressure sensors utilized in biomedical devices [62]. Conversely, in the lateral bending mode, mechanical force is applied parallel to the surface of the material, engendering a bending deformation that produces electricity. This configuration is particularly advantageous for flexible and stretchable devices, encompassing wearable sensors and textile-based energy harvesters [63].

The choice of piezoelectric materials plays a vital role in ensuring the efficiency and output capabilities of piezoelectric nanogenerators [PENGs]. These resources can be grouped into three fundamental classifications: inorganic piezoelectric materials, organic piezoelectric polymers, and hybrid nanocomposites. Inorganic materials such as zinc oxide [ZnO], lead zirconate titanate [PZT], and barium titanate [BaTiO_3_] demonstrate robust piezoelectric characteristics and remarkable durability, making them exemplary candidates for high-performance PENGs [64]. The focus on ZnO nanowires has intensified due to their impressive piezoelectric properties, simple production methods, and nanoscale adaptability [60]. Additionally, the modification of ZnO structures through doping with elements such as lithium and aluminum has been examined to enhance the energy conversion efficiency by mitigating charge screening effects and increasing polarization [60]. Polyvinylidene fluoride [PVDF] and its copolymers, classified as organic piezoelectric polymers, have garnered attention due to their lightweight characteristics, flexible mechanics, and simple processing methods [62]. PVDF exhibits pronounced piezoelectric characteristics attributed to the alignment of its molecular dipoles, which can be further enhanced through mechanical stretching and electrical poling [64]. The pliability of PVDF-based PENGs renders them particularly suitable for wearable applications, including energy-harvesting textiles, biomedical implants, and soft robotics [65]. Recent innovations have also explored bio-based organic piezoelectric materials, which offer environmentally sustainable alternatives to conventional synthetic polymers [45]. Hybrid nanocomposites that integrate both inorganic and organic piezoelectric materials present a synergistic strategy for enhancing the efficiency and mechanical properties of PENGs [61]. By combining piezoelectric nanoparticles such as ZnO, BaTiO_3_, or graphene into PVDF matrices, researchers have engineered flexible nanogenerators with an improved output voltage and durability [62]. The advent of 3D-printed hybrid nanocomposites has further facilitated the design of complex energy-harvesting structures, optimizing both the material composition and device architecture for an enhanced performance [63].

The adaptability of PENGs has led to their implementation across various domains, including wearable electronics, biomedical systems, environmental monitoring, and hybrid energy-harvesting solutions. Wearable and Biomedical Applications: One of the most promising applications of PENGs lies in self-sustaining wearable and biomedical devices. PENGs can capture biomechanical energy from human activities such as walking, breathing, and muscle contractions to power low-energy electronics, including sensors and health monitoring devices [40]. Flexible and stretchable PENGs, integrated into smart apparel, insoles, and wristbands, allow for a continuous physiological assessment without a reliance on external power sources [63]. Furthermore, implantable PENGs, like self-powered pacemakers and neural stimulators, have been explored for medical uses by harnessing energy from organ movements [65]. The development of biodegradable piezoelectric materials also improves the feasibility of temporary medical implants that can safely decompose after their functional period [38].

Environmental and Structural Health Monitoring: Piezoelectric nanogenerators [PENGs] have been widely used in autonomous environmental and structural health monitoring systems. These nanogenerators can be strategically placed within smart infrastructures to detect mechanical vibrations, changes in pressure, and material stress, thus providing real-time evaluations related to bridge integrity, seismic activity detection, and the monitoring of industrial machinery [61]. Additionally, PENGs have been incorporated into renewable energy systems to capture ambient mechanical energy from wind and water currents, facilitating sustainable energy solutions [64].

Hybrid Energy-Harvesting Systems: In an effort to enhance the overall energy conversion efficiency, hybrid energy-harvesting systems that combine piezoelectric nanogenerators [PENGs] with triboelectric nanogenerators [TENGs] and photovoltaic cells have been innovatively designed [41]. These hybrid nanogenerators enable the simultaneous harvesting of various energy sources, making them suitable for powering next-generation Internet of Things [IoT] devices, wearable electronics, and remote environmental sensing devices [58]. For example, hybrid PENG-TENG systems have been successfully deployed in self-sustaining smart textiles and sensors developed to detect human motion [67].

Despite significant advancements, numerous obstacles hinder the broad commercialization of PENG technology. A major challenge is the mechanical fatigue and degradation of piezoelectric materials when exposed to continuous stress, which can lead to a performance decline over time [52]. Additionally, improving fabrication methods to achieve the scalable and cost-effective manufacturing of flexible PENGs remains a key focus of ongoing research [38]. The integration of artificial intelligence [AI] with PENG-based sensors is expected to revolutionize real-time health monitoring and predictive diagnostics by facilitating intelligent self-powered analytics [40]. Future developments will also stress the exploration of next-generation piezoelectric nanomaterials, including two-dimensional materials, bio-inspired structures, and perovskite-based composites, to further enhance the energy conversion efficiency and mechanical strength [60]. With ongoing research efforts and technological progress, PENGs hold significant promise for advancing self-sustaining electronic systems across various fields.

### 2.3. Pyroelectric Nanogenerators

Pyroelectric nanogenerators operate based on the principles of the pyroelectric effect, which occurs in certain non-centrosymmetric materials that create an internal electric field in response to temperature changes [54]. Unlike thermoelectric nanogenerators, which rely on a stable temperature gradient, pyroelectric nanogenerators generate an electrical response due to transient temperature fluctuations [68]. This phenomenon results from the spontaneous polarization present in pyroelectric materials, where temperature changes affect the dipole orientation within the crystalline structure, leading to charge separation and the formation of an electrical potential difference [67].

**Figure 3:**
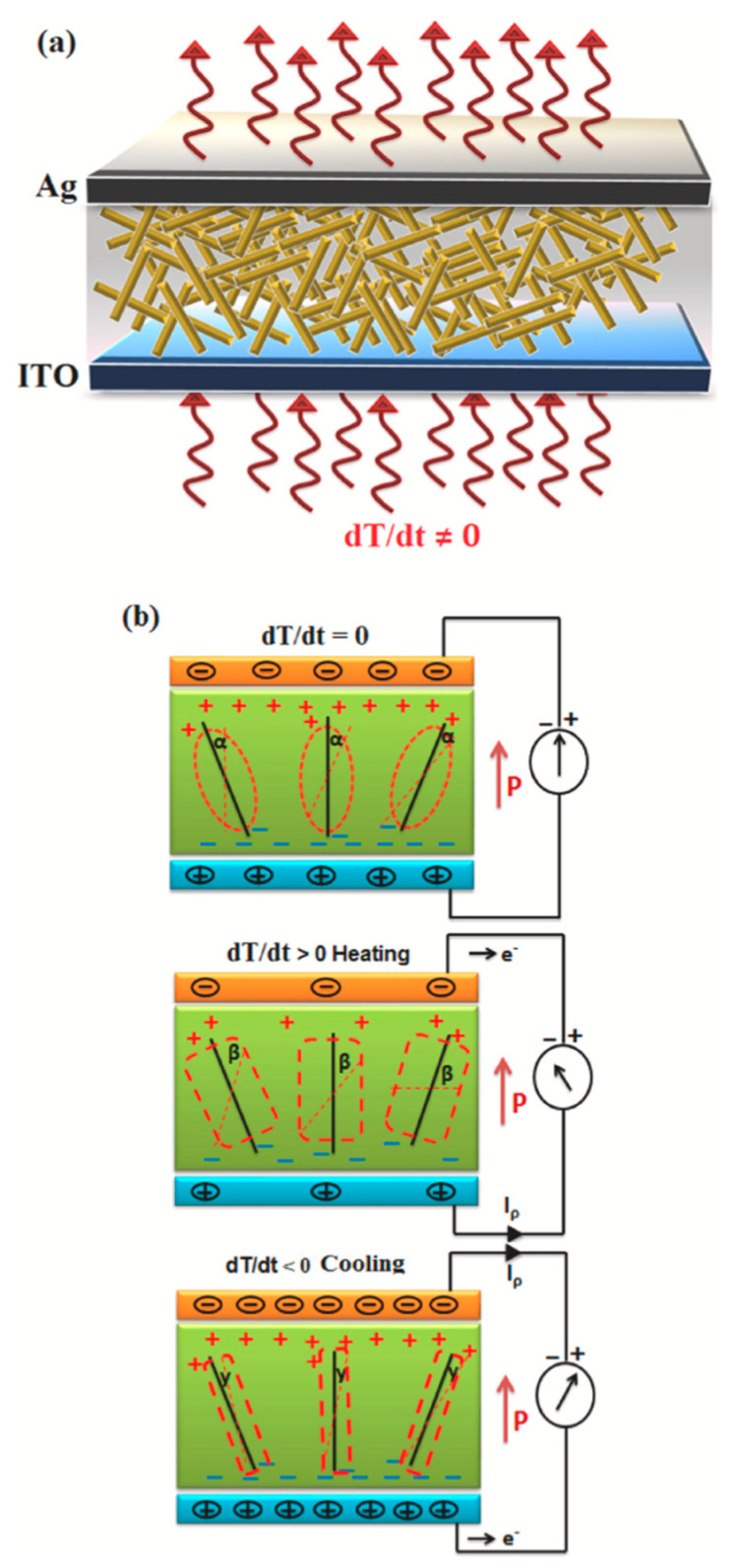
(**a**) The operational principle of PYNGs is predicated upon the pyroelectric effect. (**b**) A schematic representation of the PYNGs nanogenerator illustrating a negative electric dipole subjected to thermal cycling in a room-temperature environment [69].

When a pyroelectric material undergoes cyclical heating and cooling, the resulting dipole reorientation generates a time-varying electric current. This accumulation of charge can be effectively harnessed through electrodes and stored in capacitors or batteries to power electronic devices [55]. The working principle of the pyroelectric nanogenerator is illustrated in Figure 3. The operational efficiency of pyroelectric nanogenerators depends on the material’s intrinsic pyroelectric coefficient, the thermal variation rate, and the active layer’s surface area [58].

The judicious selection of pyroelectric materials is essential for optimizing the performance of PYNGs. The predominantly utilized pyroelectric materials include inorganic oxides, organic polymers, and hybrid nanocomposites. Inorganic Pyroelectric Materials: Lead zirconate titanate (PZT), barium titanate (BaTiO_3_), and lithium niobate (LiNbO_3_) are extensively used inorganic pyroelectric materials due to their high pyroelectric coefficients and thermal stability [70]. These materials exhibit significant polarization changes in response to thermal variations, making them exceptionally effective for energy-harvesting applications [56]. However, the brittleness and rigidity of these ceramics pose challenges for their integration into flexible and wearable electronic devices [54]. Organic Pyroelectric Materials: Organic polymers such as polyvinylidene fluoride [PVDF] and its copolymers [P[VDF-TrFE]] have gained considerable attention for their flexibility, lightweight properties, and ease of processing [67]. These materials demonstrate substantial pyroelectric responses and can be fabricated into thin films and flexible substrates, making them suitable for wearable and biomedical applications [68]. Additionally, bio-based organic pyroelectric materials have been explored for their potential in sustainable energy-harvesting solutions [55]. Hybrid Pyroelectric Nanocomposites: Hybrid pyroelectric nanocomposites combine inorganic and organic materials to improve both performance and durability. By incorporating BaTiO_3_ or ZnO nanoparticles into PVDF matrices, researchers have achieved an enhanced energy conversion efficiency and mechanical flexibility [70]. These hybrid materials enable a scalable fabrication and integration into multifunctional energy-harvesting systems [56].

Pyroelectric nanogenerators have applications across diverse domains, including waste heat recovery, biomedical instrumentation, and autonomous sensing devices. In waste heat recovery, PYNGs leverage thermal fluctuations generated by industrial operations, vehicular engines, and electronic devices to produce electrical energy, thus enhancing sustainable energy strategies [58]. In biomedical applications, PYNGs have been integrated into wearable devices that utilize intrinsic body temperature variations to power sensors and facilitate drug delivery mechanisms [67]. Furthermore, PYNGs are used in infrared detection and motion sensing technologies, where temperature-induced charge variations provide self-sustaining sensing functionalities [56].

Recent advancements in the domain of pyroelectric nanogenerators have significantly augmented their operational performance, efficiency, and applicability. Conventional challenges associated with pyroelectric devices, including a limited energy output and sluggish thermal response, have been alleviated through innovative material utilization and pioneering structural designs. Adopting materials with elevated pyroelectric coefficients, including doped barium titanate (BaTiO_3_), lithium tantalate (LiTaO_3_), and poly(vinylidene fluoride–trifluoroethylene) [P(VDF-TrFE)], has notably raised the efficiency of the energy conversion from thermal to electrical states [55]. Recent investigations have concentrated on the engineering of hierarchical structures employing advanced three-dimensional printing and templating methodologies. Architected lamellar and porous architectures have exhibited an improved capture of thermal fluctuations, thereby facilitating a superior pyroelectric current generation. To illustrate this, layered pyroelectric composites generated through direct ink writing (DIW) have revealed an increase in energy density by up to 2.5 times compared to their bulk-processed variants, achieving figures up to 1.2 mJ/cm^2^ during rapid temperature cycling [54].

Additionally, new hybrid strategies have been established that merge pyroelectric nanogenerators with triboelectric layers to produce dual-mode energy harvesters that efficiently gather both thermal and mechanical energy. Such hybrid systems effectively address the intermittent nature of temperature fluctuations prevalent in ambient environments, thereby enhancing the overall reliability and power output of the devices [49]. In the biomedical sector, pyroelectric nanogenerators have been employed as body-heat-driven power sources for implantable devices. Flexible pyroelectric nanogenerators based on P(VDF-TrFE)/BaTiO_3_ composites have been embedded into wearable patches, demonstrating a stable energy generation from minimal temperature variations of less than 2 °C, which is adequate for the continuous operation of low-power biosensors without the need for external batteries [71]. Moreover, recent developments in self-healing pyroelectric composites have facilitated extended device lifetimes. Self-repairable polymer matrices combined with pyroelectric fillers enable devices to retain their functionality even after mechanical damage, thus enhancing their appropriateness for wearable and implantable systems [52].

### 2.4. Hybrid Nanogenerators

Hybrid nanogenerators (HNGs) represent sophisticated multifunctional energy-harvesting systems meticulously engineered to synergistically amalgamate various energy conversion mechanisms within a unified framework, thereby augmenting the versatility and efficacy of energy harvesting across a multitude of operational contexts [41]. In opposition to standard nanogenerators that function through one specific mechanism, HNGs amalgamate different phenomena—including piezoelectric, triboelectric, pyroelectric, and photovoltaic effects—to concurrently gather mechanical, thermal, and solar energy. This multi-modal capability not only facilitates continuous power generation but also ensures adaptability for an extensive array of self-sustaining systems [67].

Piezoelectric–Triboelectric Hybrid Nanogenerators (PT-HNGs): Piezoelectric–triboelectric hybrid nanogenerators (PT-HNGs) represent a prominent focus of research within the domain of hybrid energy-harvesting systems, due to their remarkable capacity for harvesting mechanical energy from diverse dynamic sources, such as motion, vibration, and pressure variations [54]. These devices operate through the integration of two complementary energy conversion methods—the piezoelectric effect and the triboelectric effect, both of which facilitate the conversion of mechanical deformation into electrical energy, albeit through distinct fundamental principles.

The piezoelectric effect functions according to the intrinsic features of various dielectric materials, including polyvinylidene fluoride (PVDF), zinc oxide (ZnO), and lead zirconate titanate (PZT), resulting in electric potential when they endure mechanical force. This mechanical deformation results in a displacement of the internal electric dipoles, thereby establishing a polarization field and culminating in the accumulation of charge on the surface of the material. The resultant voltage generated is directly proportional to the applied force and the properties of the material, rendering it particularly suitable for low-frequency and intermittent motions such as ambulation, bending, or bodily movement [72].

Conversely, the triboelectric effect relies on contact electrification occurring between two different materials with varying electron affinities, such as polydimethylsiloxane (PDMS) and nylon, followed by electrostatic induction that generates a flow of electrons upon separation [67]. When these surfaces make contact and then separate, electrons move between the materials, causing charge separation and creating a potential difference. This mechanism is particularly effective in capturing micro-scale motion, repetitive contact, or sliding interactions that are common in daily human activities [54].

The confluence of these mechanisms within a PT-HNG architecture creates a synergistic interaction that significantly increases the energy output, expands the operational frequency range, and improves the adaptability to variable mechanical inputs [72]. For example, while in motion, pressure (linked with piezoelectric effects) and friction (associated with triboelectric effects) are generated simultaneously at the interface of the foot and the shoe, promoting dual-mode energy harvesting and boosting the power density compared to single-mode nanogenerators [68]. Moreover, the integration of stretchable substrates and textile-compatible materials has enabled the development of flexible and conformable PT-HNGs for applications in wearable technology and skin-integrated systems [72].

These innovative hybrid systems offer significant benefits for powering future self-sufficient electronic devices, including fitness trackers, heart-rate monitors, motion detectors, and intelligent clothing [54]. Their sensitivity to both tactile and structural deformations positions them well for interactive human–machine interfaces and real-time biomechanical feedback within healthcare systems [68]. Additionally, their use in industrial settings for machinery monitoring and in the automotive sector for tire and vibration energy recovery is rapidly growing, emphasizing their versatility and technological potential [41].

Nevertheless, PT-HNGs face technical challenges, including the need for material optimization to reduce mechanical fatigue and enhance the charge transfer efficiency [67]. Interface engineering between the piezoelectric and triboelectric layers remains a critical focus for improvement to ensure a robust and stable output under conditions of repeated cycling [73]. Future advancements are directed towards the development of integrated circuit systems for effective energy storage, self-starting mechanisms, and adaptive load matching using controllers based on artificial intelligence [58].

**Figure 4:**
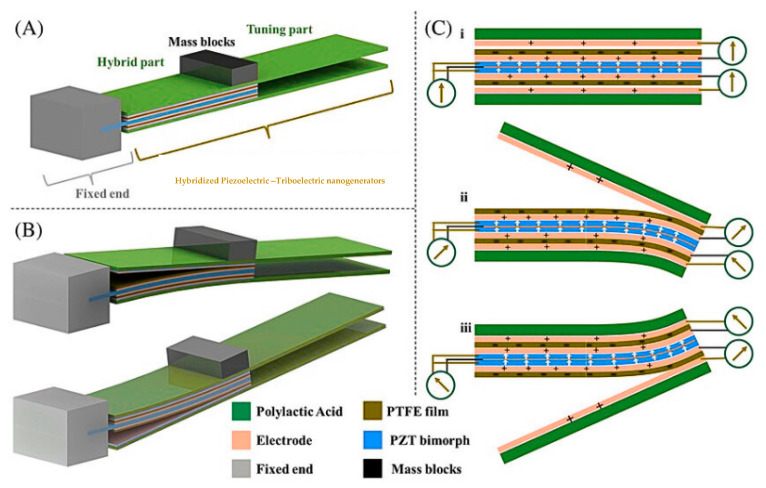
(**A**) A schematic representation of the structural configuration. (**B**) Constituent materials and underlying mechanical principles. (**C**) Operational mechanisms of the hybrid component [74].

Pyroelectric–Triboelectric Hybrid Nanogenerators (PyT-HNGs): Pyroelectric–triboelectric hybrid nanogenerators (PyT-HNGs) integrate two distinct energy conversion mechanisms—pyroelectricity and triboelectricity—facilitating the concurrent harvesting of thermal and mechanical energies from the ambient environment [56]. Figure 4 provides an integrated view of the hybrid nanogenerator’s structure, material composition, and operational mechanisms, emphasizing its multi-source energy harvesting potential. The pyroelectric segment functions on the basis of dynamic polarization phenomena that manifest in specific polar crystalline materials when subjected to temporal temperature fluctuations, which may arise from sources such as human body heat, solar radiation, or variations in ambient temperature [58]. Upon the occurrence of these thermal changes, the internal electric dipoles within the pyroelectric material undergo realignment, yielding a spontaneous polarization that culminates in a transient voltage output [56]. This operational principle is particularly advantageous in contexts where steady-state thermal conditions are supplanted by intermittent thermal inputs or outputs, exemplified by daily temperature oscillations or thermally active physiological regions.

Conversely, the triboelectric component is predicated upon the principles of contact electrification and electrostatic induction, which facilitate the conversion of kinetic energy derived from friction, tapping, or vibrational sources into usable electrical energy [54]. Usually, triboelectric substances distinguished by varying electron affinities—such as PTFE and nylon—are contacted and then separated, causing an accumulation of surface charges and a resulting potential difference. This triboelectric phenomenon provides an autonomous mode of energy harvesting that enhances the efficacy of the pyroelectric response, particularly in scenarios characterized by limited thermal inputs yet adequate mechanical motion [68].

The interplay between these dual mechanisms within PyT-HNGs results in an enhanced energy harvesting efficiency under hybrid thermal–mechanical excitation conditions. For instance, bodily motion during physical activity induces a mechanical deformation conducive to a triboelectric output while concurrently elevating body temperature, thereby instigating shifts in the pyroelectric polarization [56]. Such attributes render PyT-HNGs exceedingly appropriate for self-sustaining biomedical applications, including implantable biosensors, cardiac stimulators, and cutaneous thermal monitoring devices [68]. Furthermore, their aptitude for scavenging energy from ambient thermal sources and tactile interactions positions them as ideal candidates for environmental sensing applications within smart infrastructure and industrial process oversight [58].

Notwithstanding their significant potential, PyT-HNGs encounter challenges, including the unpredictability of thermal fluctuations in uncontrolled settings and the scarcity of high-performance pyroelectric materials that are compatible with flexible substrates [68]. Prospective advancements in the development of PyT-HNGs will likely involve the engineering of multifunctional nanocomposites, the incorporation of flexible ceramic and polymer blends, as well as the exploration of multilayer device architectures aimed at optimizing the energy yield and mechanical stability [67].

Photovoltaic–Piezoelectric Hybrid Nanogenerators (PV-PENGs): Photovoltaic–piezoelectric hybrid nanogenerators (PV-PENGs) are engineered to concurrently harness both solar and mechanical energy, thereby facilitating robust and continuous energy generation across diverse illumination and motion scenarios [70]. The photovoltaic segment transduces solar radiation into electrical energy through the photoelectric effect. In this activity, semiconductor materials, including silicon, cadmium telluride, or perovskites, absorb incoming photons, exciting electrons from the valence band to the conduction band, producing electron–hole pairs that are later separated by an internal electric field to create an electric current.

In parallel, the piezoelectric part seizes mechanical energy via strain-induced polarization in compounds such as zinc oxide (ZnO), polyvinylidene fluoride (PVDF), or lead zirconate titanate (PZT) [67]. As mechanical stress—such as vibrations, impacts, or bending—acts upon the piezoelectric material, it produces a voltage from the shift of electric dipoles in its crystalline framework. This phenomenon is particularly advantageous in wearable or mobile contexts where motion is prevalent and predictable. The integration of these two mechanisms permits PV-PENGs to remain functional even when one energy source is temporarily unavailable. For instance, in indoor environments or during overcast conditions where solar irradiance is diminished, the piezoelectric component can persist in supplying power derived from user movement or ambient vibrations [70]. Conversely, in static outdoor settings characterized by abundant sunlight but minimal movement, the photovoltaic layer guarantees uninterrupted energy harvesting. This dual mechanism facilitates seamless transitions between energy modes and amplifies the overall power output compared to systems reliant on a single energy source [58].

PV-PENGs have been employed across various domains, including intelligent building facades, wearable solar textiles, marine buoys, and space-constrained Internet of Things (IoT) devices [55]. Notably, their application in solar–textile integration paves the way for the creation of garments that generate electricity both during ambulation and in sunlight, presenting a promising avenue for off-grid energy production in remote areas [54]. Furthermore, in the realms of oceanic and environmental sensing, PV-PENGs adeptly combine wave-induced mechanical motion with solar energy, thereby sustaining the energy supply for sensors and communication systems throughout diurnal cycles [58]. A significant challenge in the advancement of PV-PENGs resides in optimizing the compatibility of materials and the mechanical durability of layered or co-integrated photovoltaic and piezoelectric configurations [67]. Interfacial energy dissipation, the optical degradation of photovoltaic materials under mechanical strain, and the challenges associated with achieving flexible and transparent arrangements impede both performance and longevity. Ongoing investigations are thus aimed at combining stretchable solar polymers with pliable piezoelectric nanomaterials, innovating transparent conductive electrodes, and employing encapsulation strategies to bolster durability in outdoor settings [41].

## 3. Nanocomposites in 3D-Printed Nanogenerators: Design and Properties

The advancement of 3D-printed nanocomposites has significantly transformed the energy harvesting field, facilitating the intricate production of versatile, energy-efficient nanogenerators suitable for wearable devices, biomedical applications, and self-powered systems. Unlike conventional nanogenerators reliant on bulk material processing techniques, additive manufacturing allows for the effortless incorporation of nanomaterials into functional devices, thereby enhancing mechanical durability, energy conversion efficiency, and structural adaptability [75,76].

Nanocomposites play a critical role in enhancing piezoelectric, triboelectric, and hybrid nanogenerators, where the interaction between polymeric matrices and functional nanofillers influences the charge transfer, flexibility, and overall device performance [77]. By combining nanofillers such as graphene, MXenes, carbon nanotubes [CNTs], metal oxides, and piezoelectric ceramics with printable polymers, we have advanced the innovation of custom energy-harvesting solutions. The versatility of 3D-printed nanocomposites includes biodegradable, wearable, and transparent nanogenerators, thus promoting progress in self-sustaining energy systems for next-generation applications [78,79].

### 3.1. Three-Dimensional-Printable Piezoelectric Nanocomposites for Nanogenerators

Piezoelectric materials have garnered significant interest in energy-harvesting applications due to their ability to convert mechanical energy into electrical energy when subjected to stress. The emergence of 3D-printable piezoelectric nanocomposites has enabled the creation of flexible, customizable, and efficient nanogenerators, thus improving their mechanical properties and versatility for various applications [80]. These printable materials provide precise control over structural parameters, optimizing the piezoelectric performance and increasing their suitability for wearable electronics, biomedical devices, and self-powered systems [81]. The ability to incorporate nanofillers, polymer matrices, and hybrid composites into printable inks further enhances the energy conversion efficiency of these nanogenerators [82].

A critical aspect of determining the effectiveness of piezoelectric materials is the d33 piezoelectric constant, which indicates the amount of electric charge created for every unit of mechanical stress imposed in the same alignment. An elevated d33 value signifies an enhanced energy conversion efficiency, rendering it an essential consideration in the formulation of piezoelectric materials for energy-harvesting purposes. Scholars have persistently investigated techniques to augment the d33 values of diverse materials through compositional alterations and nano-structuring methodologies. Advanced piezoelectric ceramics characterized by high d33 values are particularly conducive to applications including film speakers and energy-harvesting devices [83].

Subsequent investigations have demonstrated that optimizing material compositions can significantly enhance the d33 constant. Both improved piezoelectric constants and a reduced dielectric loss in BiScO3-PbTiO_3_–Bi [Mn_2/3_Sb_1/3_]O_3_ ceramics, showcasing the potential of tailored material systems for better energy harvesting outcomes [84]. CBN ceramics and incorporating Ce ions greatly enhances piezoelectric characteristics, thereby improving their efficiency in nanogenerators [85]. Additionally, in the realm of polymeric piezoelectric materials, variable piezoelectric PLLA nanofiber membranes, revealing enhanced piezoelectric properties are suitable for medical and autonomous applications [86]. Table 1 summarizes typical d33 values for various 3D-printed and conventional piezoelectric materials, along with references to notable studies.

#### 3.1.1. Polymer-Based Printable Piezoelectric Materials

##### Polyvinylidene Fluoride [PVDF] and P[VDF-TrFE]

**Figure 5:**
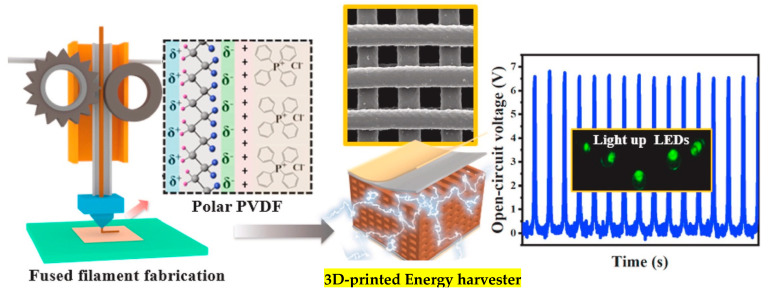
Three-dimensional printing of PVDF [95].

Polyvinylidene fluoride [PVDF] and its copolymer poly[vinylidene fluoride-trifluoroethylene] [P[VDF-TrFE]] are recognized as some of the most prevalent polymer-based piezoelectric materials due to their high piezoelectric coefficients and inherent flexibility [91]. By 3D printing inks derived from PVDF, a detailed control over polymer crystallinity can be exercised, which is crucial for enhancing the β-phase formation and optimizing the piezoelectric response [92]. The schematic representation of PVDF 3D printing is illustrated in Figure 5. Recent advancements in additive manufacturing methods have enabled the creation of complex PVDF structures with improved mechanical properties, making them exceptionally suitable for flexible energy-harvesting devices [93]. These materials have found widespread applications in fields such as wearable technology, medical implants, and autonomous sensors, owing to their compatibility with biological systems and ease of processing [94].

**Figure 6:**
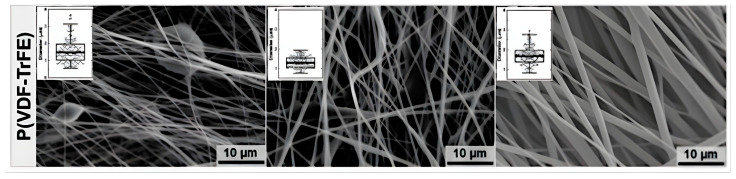
SEM images illustrating the electrospun scaffolds of PVDF and P(VDF-Trfe) derived from solutions containing varying polymer concentrations. The accompanying inserts depict the corresponding box plots [100].

The integration of P[VDF-TrFE] into 3D-printable inks has further enhanced the effectiveness of piezoelectric nanogenerators. Figure 6 displays the cross-section SEM image of P[VDF-TrFE]. This copolymer exhibits exceptional ferroelectric properties and improved piezoelectric capabilities compared to standard PVDF, making it a leading candidate for advanced nanogenerators [96]. The development of P[VDF-TrFE] composites that incorporate aerogels has led to the emergence of energy harvesters that are both stretchable and porous, demonstrating a better energy conversion efficiency [97]. This material has achieved significant applications in wearable sensors, flexible touchscreens, and implantable medical devices [98]. Moreover, fully printed P[VDF-TrFE] sensors have shown an increased sensitivity, facilitating their use in motion sensing and human–machine interfaces [99].

##### Polyurethane [PU] Composites

**Figure 7:**
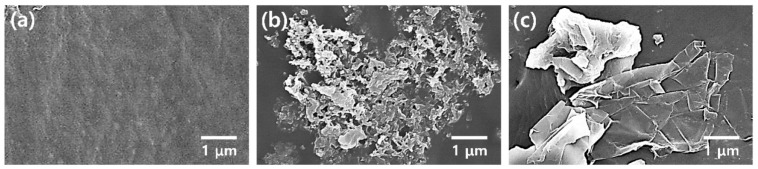
Utilizing a Field Emission Scanning Electron Microscope [FE-SEM], we observe three-dimensional-printed polyurethane [PU] composites with diverse fillers: (**a**) standard PU, (**b**) PU combined with 6 weight percent [wt%] polyaniline [PANI], and (**c**) PU blended with 2 weight percent [wt%] graphene oxide [GS] [108].

Polyurethane [PU]-based piezoelectric composites have emerged as a compelling alternative due to their exceptional mechanical strength and elasticity, which makes them highly suitable for applications in wearable and stretchable electronics [101]. The integration of PU with piezoelectric fillers, such as polyvinylidene fluoride [PVDF] or ceramic nanoparticles, enables the creation of highly flexible energy harvesters that exhibit outstanding mechanical durability [102]. These materials have been utilized in self-sustaining sensing applications, including pressure sensors and biomedical devices [103]. The SEM images of the 3D-printed PU composite are presented in Figure 7.

Recent advancements in multi-material three-dimensional printing technologies have propelled the development of PU-based hybrid nanogenerators with tunable electrical properties [104]. These composites are particularly beneficial for applications in soft robotics, electronic skin, and healthcare monitoring systems [105]. The stimuli-responsive characteristics of PU composites, when combined with liquid metal hybrids, have further expanded their applicability in human-interactive electronics and wearable bioelectronics [106].

The potential of polymer-derived printable piezoelectric materials is significant for upcoming energy harvesting advancements. The ability to integrate multiple functionalities within three-dimensional-printed nanogenerators enables the creation of highly efficient, lightweight, and versatile energy harvesters for future technological innovations [107]. Anticipated future research efforts are expected to focus on enhancing the electromechanical coupling efficiency of printable piezoelectric materials while maintaining flexibility and durability for practical applications in real-world scenarios.

##### Polydimethylsiloxane [PDMS]

Polydimethylsiloxane [PDMS], an elastomeric polymer extensively employed in various applications, is distinguished by its remarkable flexibility, biocompatibility, and mechanical integrity, rendering it particularly advantageous for the fabrication of 3D-printed triboelectric nanogenerators [TENGs]. Its intrinsic capacity to generate triboelectric charges through contact with diverse materials has established it as an essential component in wearable and stretchable energy-harvesting devices [78]. The low surface energy characteristic of PDMS significantly enhances triboelectric phenomena, especially when micro- and nanoscale surface texturing is implemented through advanced 3D printing methodologies, such as direct ink writing [DIW], extrusion-based printing, and Digital Light Processing [DLP] [109]. Moreover, its high transparency coupled with its resistance to environmental degradation positions it as an optimal choice for long-term energy-harvesting applications [110]. PDMS composite materials suitable for 3D printing, enhanced with conductive nanofillers like carbon nanotubes [CNTs], graphene, and MXenes, exhibit an impressive charge storage and electrical conductivity, boosting their triboelectric and piezoelectric features [111]. These composites have been utilized in the development of wearable, stretchable, and implantable nanogenerators designed for self-powered sensors and biomedical applications [79]. The inherent softness and stretchability of PDMS facilitate its effective processing via 3D printing, allowing for the creation of bespoke TENG structures with an optimized charge collection efficiency and mechanical resilience under repetitive deformation [112].

**Figure 8:**
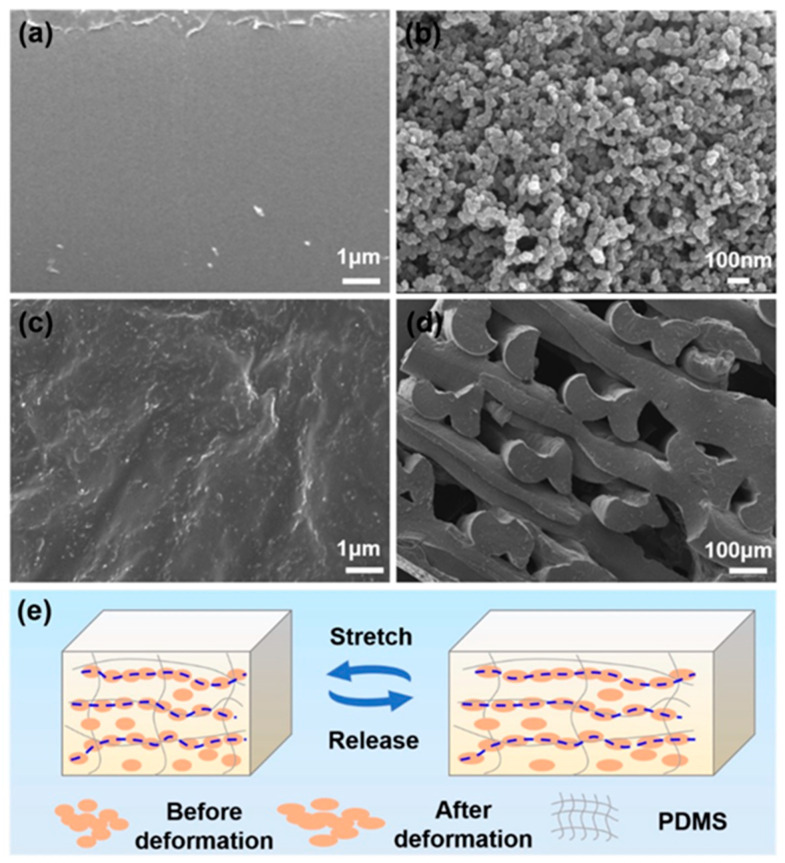
(**a**) Pure polydimethylsiloxane [PDMS]; (**b**) a Scanning Electron Microscopy [SEM] image displaying carbon black particles; (**c**) the composite material made of carbon black and polydimethylsiloxane [CB/PDMS]; (**d**) the structural arrangement of the three-dimensional-printed composite; and (**e**) the change in the conductive pathway of the CB/PDMS composite when subjected to mechanical stress [83].

Recent research endeavors have concentrated on 3D-printed PDMS-based porous structures, wherein the enhanced surface area and charge retention substantially elevate the triboelectric energy output [113]. Figure 8 shows the SEM images of 3D-printed PDMS. The capacity to meticulously adjust the material composition and structural design through additive manufacturing affords unparalleled control over the mechanical characteristics, surface topographies, and electrical performance, establishing PDMS-based 3D-printed nanogenerators as a seminal advancement in self-powered electronic systems.

##### Thermoplastic Polyurethane [TPU]

**Figure 9:**
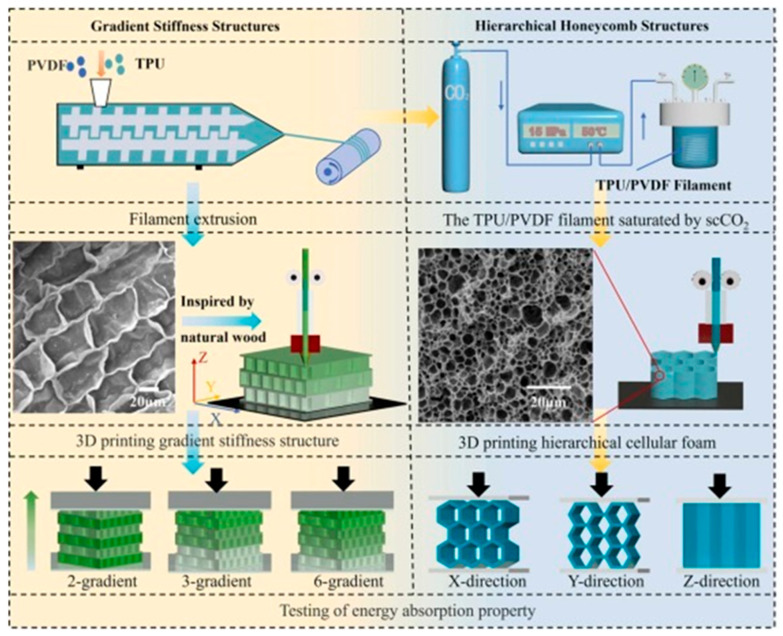
A schematic representation of a three-dimensional-printed gradient stiffness structure composed of thermoplastic polyurethane and polyvinylidene fluoride, exhibiting a hierarchical honeycomb configuration [84].

TPU is particularly valuable for wearable energy-harvesting technologies due to its impressive stretch capacity and ability to withstand repeated mechanical stress while maintaining a consistent energy output [114]. The schematic representation of 3D-printed TPU/PVDF is shown in Figure 9. The triboelectric characteristics of TPU can be optimized by incorporating high-permittivity nanofillers, which significantly enhance the charge retention and dielectric behavior [115].

In the context of 3D-printed triboelectric nanogenerators [TENGs], TPU is valued for its high electron affinity, durability, and surface versatility, which enable the creation of customized and effective charge generation architectures [116]. TPU-based TENGs have been successfully used in autonomous motion sensors, soft robotics, and wearable electronic devices, demonstrating their ability to maintain their performance across various substrates and environmental conditions [117]. The incorporation of nanofiller-reinforced TPU composites into 3D-printable inks has further improved the energy conversion efficiency of these nanogenerators [118].

An important perk of TPU arises from its integration with various 3D printing methods, such as fused deposition modeling [FDM], selective laser sintering [SLS], and direct ink writing [DIW]. This integration enables the creation of unique, lightweight, and high-performance energy-collecting frameworks [119]. The meticulous control over the material composition and structural design allows TPU-based nanogenerators to be tailored precisely to meet specific mechanical and electrical requirements. Consequently, this fosters the development of next-generation self-powered devices characterized by enhanced wearability, flexibility, and durability.

##### Ecoflex

Ecoflex, a biodegradable and highly flexible silicone-based elastomer, has garnered substantial scholarly interest in the field of 3D-printed energy-harvesting technologies due to its exceptional stretchability, softness, and biocompatibility [120]. Its widespread use in wearable and implantable nanogenerators is due to its ability to conform effortlessly to human skin and soft tissues, making it an ideal material for biomedical and physiological monitoring applications [121]. The mechanical resilience exhibited by Ecoflex allows it to endure extreme levels of deformation without experiencing structural failure, establishing it as a reliable option for flexible energy-harvesting devices [122].

In the context of 3D-printed triboelectric nanogenerators [TENGs], Ecoflex exhibits a low modulus and superior surface adaptability, which together enhance the efficiency of the triboelectric charge transfer through an optimized material contact [123]. Various additive manufacturing techniques, such as extrusion-based printing and vat photopolymerization, have been used to fabricate Ecoflex-based TENGs with tunable surface architectures, thereby significantly boosting their energy-harvesting capabilities. Recent studies have also explored the reinforcement of Ecoflex with conductive nanomaterials, including MXenes, carbon nanotubes [CNTs], and silver nanowires, which further improve its electrical characteristics and overall energy conversion efficiency [124].

**Figure 10:**
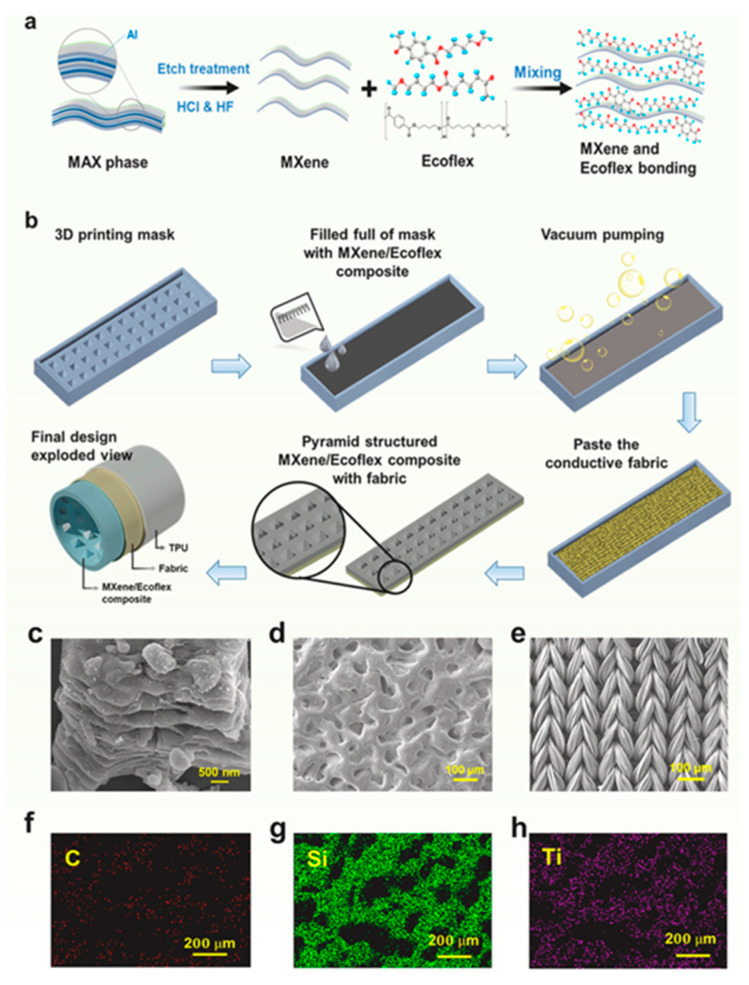
(**a**) The methodology for the fabrication of Mxene/Ecoflex nanocomposites. (**b**) The procedural framework for the development of a self-powered toroidal triboelectric sensor [STSS]. (**c**) Field Emission Scanning Electron Microscopy [FESEM] imagery depicting Mxene nanosheets. (**d**) Field Emission Scanning Electron Microscopy [FESEM] imagery illustrating Mxene/Ecoflex nanocomposites. (**e**) A conductive textile material. (**f**–**h**) Energy dispersive X-ray spectroscopy of Carbon, silicon, and titanium distribution with respect to Mxene/ecoflex composite [85].

Furthermore, hybrid energy harvesters that integrate Ecoflex-based piezoelectric and triboelectric components have been explored for multimodal energy harvesting from various mechanical stimuli, including human motion, vibrations, and biomechanical signals [125]. The biodegradability and compatibility of Ecoflex with sustainable electronics and smart textiles highlight its potential for developing next-generation self-powered systems, where flexibility, biocompatibility, and environmental sustainability are of the utmost importance [126]. Figure 10 illustrates the synthesis of MXene via the selective etching of aluminum from the MAX phase using HF and HCl. It also presents the molecular structure of Ecoflex, MXene nanosheets, and their binding in the nanocomposite. Additionally, it depicts the fabrication process of MXene/Ecoflex nanocomposites with a pyramidal structure, emphasizing their gel-state properties before drying, which allows for good flow and immersion.

Utilizing advancements in 3D printing, unique Ecoflex-derived energy harvesters can be crafted for uses in wearable tech, medical implants, and flexible robotics, offering efficient, versatile, and eco-friendly energy alternatives for upcoming autonomous devices.

#### 3.1.2. Ceramic-Based Printable Piezoelectric Materials

Among 3D-printed nanogenerators, ceramic-derived piezoelectric materials are extensively employed and celebrated for their impressive piezoelectric features, improved thermal strength, and notable mechanical properties. This avenue of inquiry has comprehensively delved into the attributes of barium titanate [BaTiO_3_] and zinc oxide [ZnO] nanowires with respect to their effectiveness in raising the energy conversion efficiency of piezoelectric nanogenerators [127]. The integration of these ceramic materials into printable matrices facilitates the creation of customizable, flexible, and high-performance devices for energy harvesting [128].

##### Barium Titanate [BaTiO_3_] Nanoparticles

**Figure 11:**
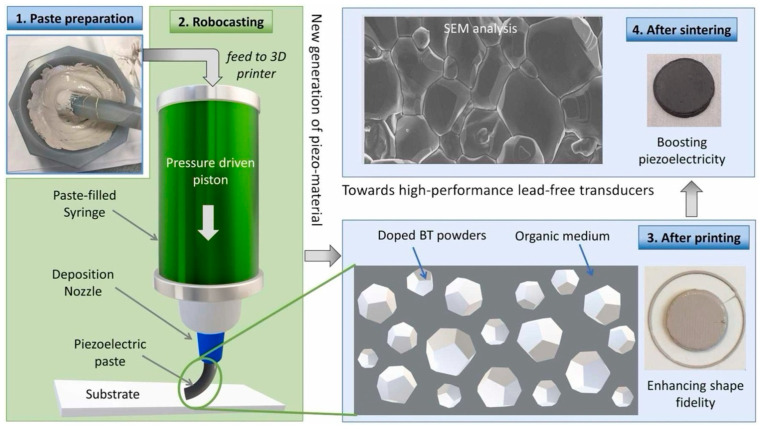
Three-dimensional printing of doped barium titanate [127].

Barium titanate [BaTiO_3_] is a lead-free piezoceramic material known for its very high dielectric constant and strong ferroelectric properties, making it a highly favorable candidate for producing three-dimensional-printed piezoelectric nanogenerators [127]. Figure 11 illustrates the 3D printing of doped barium titanate using robocasting. The material’s inherent ability to generate an electric charge upon mechanical deformation has led to its incorporation in printable polymer composites, such as polyvinylidene fluoride [PVDF] and polydimethylsiloxane [PDMS], aimed at enhancing the overall energy generation of nanogenerators [129]. The introduction of robocasting methods for BaTiO_3_-infused nanogenerators has facilitated the creation of structures with significant tunability, thereby enhancing the piezoelectric performance [130]. Additionally, inks made from BaTiO_3_ nanoparticles have been successfully utilized in direct ink writing [DIW] and extrusion-based three-dimensional printing techniques to produce flexible, high-performance energy-harvesting devices [131]. These nanogenerators are particularly suitable for autonomous Internet of Things [IoT] devices, where efficient energy conversion is crucial for the functioning of tiny sensors and electronic components [128].

##### Zinc Oxide [ZnO] Nanowires

Zinc oxide [ZnO] nanowires have attracted considerable scholarly interest as a printable piezoelectric material due to their unique electromechanical coupling and excellent piezoelectric properties [80]. Compared to bulk ceramics, ZnO nanowires demonstrate remarkable flexibility, making them suitable for use in flexible and stretchable nanogenerators employed in self-sustaining electronic devices [132]. ZnO-based piezoelectric nanogenerators have been produced using inkjet printing and direct ink writing [DIW] techniques, thus enabling the creation of highly customizable energy-harvesting devices [133]. The application of ZnO nanowires onto soft polymer substrates, such as PDMS and polyurethane, fosters the development of wearable and implantable piezoelectric technologies, particularly in the biomedical field [134].

Incorporating ZnO nanowires into three-dimensional-printed frameworks significantly enhances the charge transfer efficiency, resulting in an increased energy output [135]. Moreover, the site-selective growth of ZnO nanowires has been proven to be an effective strategy for optimizing their orientation and alignment, thereby enhancing piezoelectric responsiveness [133]. These advancements have enabled the creation of self-powered biosensors and biomedical implants capable of continuously monitoring physiological signals without a dependence on external power sources [136].

The advancement of three-dimensional-printed ceramic-based piezoelectric materials, particularly BaTiO_3_ and ZnO nanowires, has significantly propelled the field of energy harvesting forward. The ability to print these materials into tailored and flexible shapes has enabled the fabrication of high-performance nanogenerators suitable for a diverse array of sectors, including wearable electronics, biomedical sensors, and autonomous IoT devices [80]. Future research will continue to explore innovative printing methodologies and material formulations to further enhance the efficiency and scalability of these piezoelectric nanogenerators [128].

### 3.2. Printable Triboelectric Materials for Nanogenerators

#### 3.2.1. Graphene and Graphene Derivatives

**Figure 12:**
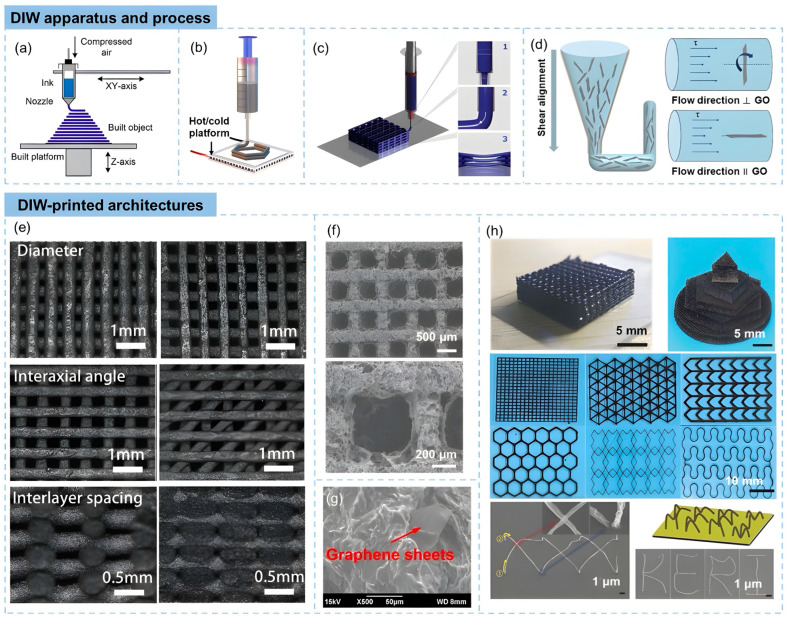
Three-dimensional printing of graphene utilizing direct ink writing [DIW] methodologies. (**a**) A schematic representation of the DIW process, (**b**) the application of DIW on a thermally regulated platform, and (**c**) the principal stages of the DIW procedure: the movement of ink within the syringe barrel and nozzle, the expulsion of ink from the nozzle, and the placement onto the substrate to create a self-supporting structure. Furthermore, (**d**) the alignment of graphene sheets induced by shear forces during the DIW procedure, (**e**) patterned constructs characterized by varying filament diameters, interaxial angles, and interlayer spacings, (**f**) microstructural features of filaments within DIW-fabricated graphene aerogel lattices, (**g**) graphene/polymer composite materials, and (**h**) architectures produced via DIW are depicted [86].

Graphene, a two-dimensional nanomaterial constituted by a singular layer of carbon atoms organized in a hexagonal lattice, has garnered considerable scholarly interest in the advancement of 3D-printable triboelectric nanogenerators [TENGs] owing to its remarkable electrical conductivity, mechanical robustness, and elevated surface area. These characteristics render graphene optimal for augmenting the charge transport and energy conversion efficiency in printed TENGs, particularly for applications necessitating flexible, lightweight, and high-performance energy-harvesting systems. The integration of graphene into 3D-printable polymer-based inks facilitates the creation of customizable and structurally complex triboelectric layers, which promote enhanced charge transfer dynamics and a superior triboelectric output [79]. In contrast to traditional TENG fabrication methodologies, 3D printing utilizing graphene-based materials affords meticulous control over the material composition and structural design, culminating in an optimized charge generation and mechanical durability [112]. Figure 12 shows the schematic representation of the 3D printing of graphene.

Beyond pure graphene, its derivatives, including graphene oxide [GO] and reduced graphene oxide [rGO], have exhibited substantial potential in 3D-printed TENGs due to their adjustable electrical properties and ability to enhance triboelectric charge separation. The oxygen-rich functional groups present in GO promote a superior dispersion within printable polymer matrices, resulting in improved interfacial adhesion and charge trapping capabilities [113]. This feature is particularly advantageous for inkjet and direct ink writing [DIW] 3D printing methods, where the uniform distribution of functionalized nanomaterials is crucial for achieving high-performance printed TENGs. Furthermore, rGO, derived from the reduction of GO, exhibits an increased conductivity while maintaining flexibility, making it an appealing candidate for wearable and stretchable triboelectric nanogenerators that require a balance between electrical efficiency and mechanical compliance [137].

Recent developments have also explored hybrid graphene-based printable composites, in which graphene is combined with other functional nanomaterials such as MXenes, metal oxides, or carbon nanotubes [CNTs] to further enhance the electrical output and mechanical versatility of 3D-printed TENGs. The synergistic interactions between graphene and these additional nanofillers result in an improved electron transport, increased surface charge density, and enhanced mechanical resilience, making them suitable for multifunctional and self-powered energy systems [86]. Moreover, graphene-reinforced elastomeric composites have been investigated for stretchable TENG applications, where the material’s flexibility guarantees consistent energy harvesting even under dynamic deformation conditions such as bending, stretching, and twisting. This characteristic is particularly relevant for wearable electronics, biomedical sensors, and self-powered Internet of Things [IoT] devices, where maintaining a stable energy output during continuous movement is crucial.

Moreover, advancements in 3D printing methodologies—such as stereolithography [SLA], Digital Light Processing [DLP], and extrusion-based printing—have facilitated the scalable and high-resolution fabrication of graphene-enhanced TENG structures with customized surface morphologies. Micropatterned and porous architectures established through additive manufacturing further enhance the charge trapping and frictional contact efficiency, thus improving the triboelectric performance of printed devices [112]. By harnessing the versatility of graphene-based 3D-printable materials, researchers are pioneering next-generation TENGs that combine structural complexity, superior charge transfer, and mechanical robustness, thereby paving the way for advanced energy-harvesting solutions in smart textiles, human-motion energy harvesting, and self-powered electronic skins.

#### 3.2.2. Carbon Nanotubes

**Figure 13:**
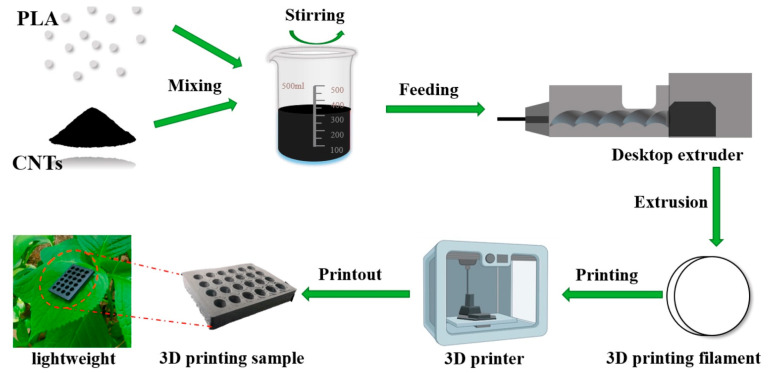
Preparation process of CNT/PLA composites [87].

Carbon nanotubes [CNTs] have emerged as a focal point in the field of nanotechnology, recognized as one of the most thoroughly examined nanofillers for 3D-printable nanogenerators due to their exceptional electrical conductivity, high aspect ratio, and superior mechanical properties. The preparation process for 3D-printed CNTs is illustrated in Figure 13. These elements are primarily classified into two main varieties—single-walled carbon nanotubes [SWCNTs] and multi-walled carbon nanotubes [MWCNTs]—which exhibit outstanding charge transport characteristics when incorporated into 3D-printable polymer-based matrices [88]. Their intrinsic flexibility and ability to form percolative conductive networks make them highly effective in enhancing charge generation and transport mechanisms in both piezoelectric nanogenerators [PENGs] and triboelectric nanogenerators [TENGs] [78].

In 3D-printed triboelectric nanogenerators [TENGs], CNTs play a crucial role in enhancing the charge transfer efficiency and surface charge density by providing additional pathways for electron migration. The incorporation of 3D-printable triboelectric polymers, such as polydimethylsiloxane [PDMS] and polyvinylidene fluoride [PVDF], with CNTs significantly improves dielectric properties and charge retention, resulting in a better triboelectric output [109]. Furthermore, CNT-reinforced 3D-printable elastomers offer essential mechanical flexibility and durability, which are vital for wearable and stretchable TENG applications. By utilizing additive manufacturing techniques, like extrusion-based 3D printing and direct ink writing [DIW], complex CNT-based TENG structures can be created with a high structural precision, enabling an adjustable surface roughness and increased frictional contact efficiency for optimized energy harvesting [78].

In the context of 3D-printable piezoelectric generators [PENGs], carbon nanotubes [CNTs] serve as supportive components that enhance stress management and structural integrity within printable piezoelectric formulations, including PVDF, P[VDF-TrFE], and polyurethane [PU] mixtures [110]. The incorporation of CNTs boosts electrical conductivity and mechanical flexibility, making them highly suitable for flexible, self-powered devices. Notably, CNT-PVDF-based 3D-printable inks have been developed to encourage β-phase crystallization, thus increasing the piezoelectric output of the produced nanogenerators. Additionally, CNT-reinforced polymer–ceramic hybrid composites have been explored for 3D-printed high-performance PENGs, where CNTs act as conductive pathways between piezoelectric particles, thereby improving the efficiency of the electromechanical energy conversion [111].

CNT-based hybrid composites have undergone extensive investigation to further enhance the energy conversion efficiency of 3D-printed nanogenerators. The combination of CNTs with functional nanomaterials, such as zinc oxide [ZnO], barium titanate [BaTiO_3_], MXenes, and graphene, leads to synergistic improvements in the charge storage, mechanical flexibility, and electrical conductivity. In 3D-printed hybrid TENGs, CNT-MXene composites have shown significant potential for increasing the triboelectric charge density, while CNT-ZnO and CNT-BaTiO_3_ composites have demonstrated superior piezoelectric responses in 3D-printed PENGs [111]. Additionally, CNT-functionalized porous scaffolds created through stereolithography [SLA] and Digital Light Processing [DLP] have been used to develop high-performance nanogenerators characterized by optimized mechanical and electrical properties.

### 3.3. Metal- and Metal Oxide-Based 3D-Printable Materials

#### 3.3.1. MXenes

MXenes, which represent a pioneering category of two-dimensional transition metal carbides, nitrides, and carbonitrides, have emerged as highly beneficial nanofillers for 3D-printable nanogenerators due to their excellent electrical conductivity, hydrophilic nature, and impressive charge storage capacity. Their unique layered structure, consisting of alternating metal and carbon/nitrogen layers, facilitates efficient charge transport and strong interfacial bonding when incorporated into 3D-printable polymer matrices, making them particularly well suited for advanced energy-harvesting devices [138].

In the study of 3D-printed triboelectric nanogenerators [TENGs], MXenes significantly enhance the surface charge density and triboelectric performance by facilitating rapid electron transfer and optimizing dielectric properties. Polymer inks suitable for 3D printing that are infused with MXene, such as polydimethylsiloxane [PDMS] and polyvinylidene fluoride [PVDF], demonstrate an improved triboelectric output by forming conductive percolation networks and carefully engineered surface microstructures, which are essential for maximizing the frictional contact efficiency [139]. Utilizing techniques like direct ink writing [DIW], extrusion printing, and screen printing enables the fabrication of MXene-based nanocomposites into customized TENG structures, featuring adjustable surface textures that enhance the energy harvesting performance.

Within the realm of 3D-printed piezoelectric nanogenerators [PENGs], MXenes serve as support materials, thereby enhancing both the mechanical robustness and polarization behavior of printable piezoelectric polymers, such as PVDF and P[VDF-TrFE]. The increased dielectric constant and conductivity of MXenes support better dipole alignment within the polymer matrix, thus increasing the piezoelectric output of printed PENGs [94]. Furthermore, the hybridization of MXenes with graphene or carbon nanotubes [CNTs] has been explored for 3D-printable PENG and TENG applications, yielding superior charge transport characteristics and highly efficient nanogenerator architectures [140]. The ability to precisely tailor MXene-based ink formulations for additive manufacturing allows for the creation of flexible, lightweight, and highly efficient energy harvesters, making them perfect for wearable and implantable self-powered devices.

#### 3.3.2. Metal Oxides

Zinc oxide [ZnO], titanium dioxide [TiO_2_], barium titanate [BaTiO_3_], and lead zirconate titanate [PZT] function as key functional nanofillers within 3D-printable nanocomposite inks owing to their superior dielectric constants, formidable piezoelectric properties, and exceptional resistance to chemical deterioration. These attributes are integral for elevating the capability of 3D-printed nanogenerators, specifically concerning piezoelectric nanogenerators [PENGs] and mixed triboelectric–piezoelectric nanogenerator systems [140].

In the realm of 3D-printable piezoelectric materials, ZnO nanoparticles and nanowires are commonly utilized due to their inherent piezoelectric properties and compatibility with flexible polymer matrices. ZnO-based 3D-printable inks, often used in conjunction with extrusion printing, inkjet printing, and stereolithography [SLA] techniques, have been formulated for applications requiring flexible and stretchable PENGs, where the reinforcement of ZnO nanoparticles significantly enhances the energy conversion efficiency [141]. Barium titanate [BaTiO_3_], recognized as a leading ferroelectric material, is another frequently used 3D-printable nanofiller that substantially increases the piezoelectric coefficient of polymer-based nanocomposites. BaTiO_3_–polymer hybrid inks, carefully developed for additive manufacturing, have demonstrated high-performance energy-harvesting capabilities, especially in applications that demand high sensitivity and mechanical durability [100].

Focusing on 3D-printed triboelectric nanogenerators [TENGs], researchers have investigated metal oxides like TiO_2_ and BaTiO_3_ for their promise in improving the retention of the triboelectric surface charge and optimizing dielectric characteristics. Through the incorporation of metal oxide nanostructures into printable polymer matrices, researchers have succeeded in achieving an enhanced charge separation efficiency and increased mechanical resilience in TENG devices. Moreover, studies have delved into hybrid composites combining metal oxides and graphene or carbon nanotubes [CNTs] for energy-harvesting platforms that are 3D-printable, resulting in the emergence of versatile, self-sufficient systems appropriate for wearable technology, medical applications, and IoT devices [114]. In a related direction, magnetically responsive nanocomposites, such as PETG–ABS–Fe_3_O_4_ systems, have demonstrated an outstanding remotely actuated shape-memory performance using 3D and 4D printing, expanding the multifunctional scope of printable materials for active electronic and energy-harvesting devices [142].

To recap, the amalgamation of metal oxides with 3D-printable materials has significantly altered nanogenerator fabrication, fostering the emergence of highly personalized, efficient, and scalable energy-harvesting mechanisms. The evolution of functional ink formulations paired with cutting-edge additive manufacturing techniques makes 3D-printed nanogenerators formed from metal oxides critical players in the future of self-powered electronic innovations and intelligent sensing applications.

### 3.4. Three-Dimensional-Printable Pyroelectric Nanocomposites for Nanogenerators

The emergence of 3D printing technology has fundamentally transformed the development of nanogenerators by enabling the precise fabrication of pyroelectric nanocomposites with customized characteristics. Pyroelectric materials, which generate an electrical charge in response to temperature fluctuations, are essential for energy-harvesting efforts, particularly in the areas of self-sustaining sensors and biomedical devices [143]. The integration of these materials into nanocomposites through advanced additive manufacturing techniques has significantly enhanced their functional properties, leading to an improved energy conversion efficiency and mechanical flexibility [144].

Lead zirconate titanate [PZT] and barium titanate [BaTiO3] are prominent among the pyroelectric materials often utilized, showcasing remarkable efficiency when incorporated into polymer matrices. The process of 3D printing facilitates the production of ultrahigh aspect ratio PZT nanostructures, which exhibit superior pyroelectric responses and are well suited for applications involving nano-Newton force sensing [145]. Furthermore, innovative multi-material 3D printing methods, like microwave-assisted fabrication, have been employed to construct architected lamellar pyroelectric generators that feature enhanced energy-harvesting capabilities [146].

A key advantage of 3D-printable pyroelectric nanocomposites lies in their ability to be customized for specific applications, including wearable and flexible electronics. The combination of innovative materials, such as conductive nanofillers and polymer blends, enables the creation of bendable and elastic energy capture devices that maintain excellent efficiency despite mechanical changes [147]. Furthermore, incorporating architected shell-based ferroelectric metamaterials through 3D printing has allowed for the programmable adjustment of pyroelectric properties, resulting in an enhanced performance in intelligent sensing applications [148].

The latest developments in triboelectric nanogenerators have benefited from the incorporation of 3D-printed pyroelectric nanocomposites. The formulation of novel polymer-based 3D-printed materials has enhanced the charge retention and output voltage, thereby making these devices more efficient for sustainable energy harvesting [149]. Additionally, additively manufactured biomedical energy harvesters that leverage pyroelectric nanocomposites have shown promise for powering implantable medical devices and wireless biosensors, thus paving the way for next-generation self-sustaining electronic systems [150].

The collaboration between 3D printing and pyroelectric nanocomposites has opened up new possibilities for generating ideas and advancing top-tier nanogenerators. As research in this area progresses, we anticipate that improvements in material components, structural designs, and manufacturing techniques will enhance the application of these energy-extracting devices across various industries, including healthcare, environmental monitoring, and portable electronics [151].

### 3.5. Biocompatible and Sustainable Nanocomposites

#### 3.5.1. Nanocellulose

Nanocellulose, sourced from natural cellulose fibers, has gained recognition as a sustainable and biocompatible nanofiller for 3D-printed nanogenerators due to its remarkable mechanical strength, biodegradability, and low density. Available in various forms—cellulose nanocrystals [CNCs], cellulose nanofibrils [CNFs], and bacterial nanocellulose—nanocellulose demonstrates exceptional structural and functional qualities, making it highly suitable for energy-harvesting initiatives [152]. Its renewable source and non-toxic characteristics set it apart as an environmentally friendly alternative to traditional synthetic nanomaterials in the development of next-generation sustainable energy devices.

The incorporation of nanocellulose into 3D-printable polymer matrices significantly enhances both mechanical integrity and functional adaptability, thereby rendering it highly appropriate for the fabrication of customized, flexible, and high-performance nanogenerators. The broad surface area and moisture-absorbing characteristics of nanocellulose assist in creating solid interfacial bonds with conductive and piezoelectric nanofillers, like metal oxides and carbon nanotubes [CNTs], resulting in hybrid nanocomposites that demonstrate a higher energy conversion efficiency [119]. In triboelectric nanogenerators [TENGs], nanocellulose-based 3D-printed structures demonstrate an exceptional triboelectric performance, primarily due to their ability to maintain surface charges and enhance the contact electrification efficiency [84]. The ability to carefully design and control nanocellulose-based architectures using 3D printing techniques—such as extrusion-based bioprinting and stereolithography [SLA]—enables the development of bio-inspired, porous, and lightweight TENGs with improved charge generation capabilities.

Furthermore, advancements in nanocellulose-enhanced inks have facilitated the development of fully compostable, 3D-printed energy collectors, paving the way for autonomous wearable devices, implantable medical instruments, and sustainable energy innovations. The ability to modify the mechanical, electrical, and triboelectric properties of composites printed in 3D with nanocellulose demonstrates their potential as a next-generation, eco-friendly, and superior material for nanogenerator applications.

#### 3.5.2. Silk Fibroin and Chitosan-Based Composites

Silk fibroin [SF] is a protein polymer derived from nature, known for its excellent mechanical resilience, flexibility, and biocompatibility, making it especially suitable for the production of three-dimensional-printed nanogenerators [153]. The use of silk fibroin-based bioinks has become common in the manufacturing of triboelectric nanogenerators [TENGs] and biomedical energy collectors, resulting in stable, conductive, and environmentally friendly materials [154]. The combination of silk fibroin with various nanomaterials, including silica, propolis, and metallic nanoparticles, has been studied to enhance the electrical conductivity and antibacterial properties of these devices [153]. Furthermore, SF-derived triboelectric nanogenerators [TENGs] have been effectively utilized in healthcare monitoring systems and implantable biomedical devices due to their sustainable and biocompatible characteristics [154].

Recent findings have demonstrated that silk fibroin materials can be effectively utilized for 3D printing and developed into hybrid aerogels for biocompatible and antibacterial uses, thereby widening their scope in wearable and implantable biomedical electronics [153]. In addition, SF-based nanogenerators combined with biodegradable substrates have exhibited considerable promise in the realm of self-powered biosensors for clinical applications [155].

**Figure 14:**
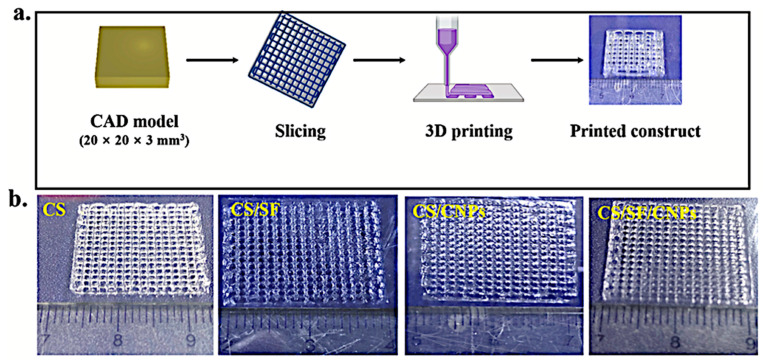
(**a**) A schematic illustration delineating the methodology for the fabrication of a three-dimensional construct composed of a chitosan/silk fibroin nanocomposite. (**b**) Visual representations of the synthesized scaffolds [88].

Employing 3D-printed silk fibroin with chitosan-based composites in biomedical electronics and eco-friendly sensing tools has sparked the development of innovative sustainable energy-harvesting solutions [155] (Figure 14). Utilizing these compostable frameworks, triboelectric nanogenerators [TENGs] have been seamlessly integrated into autonomous health monitoring systems, featuring on-body biosensors, strain sensing technology, and electronic devices designed for implantation [156]. Additionally, materials combining silk fibroin with chitosan have been applied in contexts involving electromagnetic interference [EMI] shielding, highlighting their multifunctional properties in sustainable bioelectronics [157].

Moreover, bioinks derived from chitin and chitosan have become noteworthy materials for the development of flexible electronics via 3D printing, thanks to their potential to yield structures that are not only highly conductive but also capable of extension, working well for nanogenerator uses [158]. The biodegradable and non-toxic characteristics of these materials guarantee their secure incorporation into clinical and biomedical contexts, rendering them exemplary candidates for implantable energy-harvesting systems [156].

Table 2 presents a comparative overview of the major materials discussed, summarizing their respective strengths and limitations relevant to 3D-printed nanogenerator applications. Table 3 presents recent case studies of 3D-printed nanogenerators. The table includes the material system, 3D printing technique, type of nanogenerator, its applications, and output performance.

## 4. Performance Enhancement of 3D-Printed Nanocomposites

**Figure 15:**
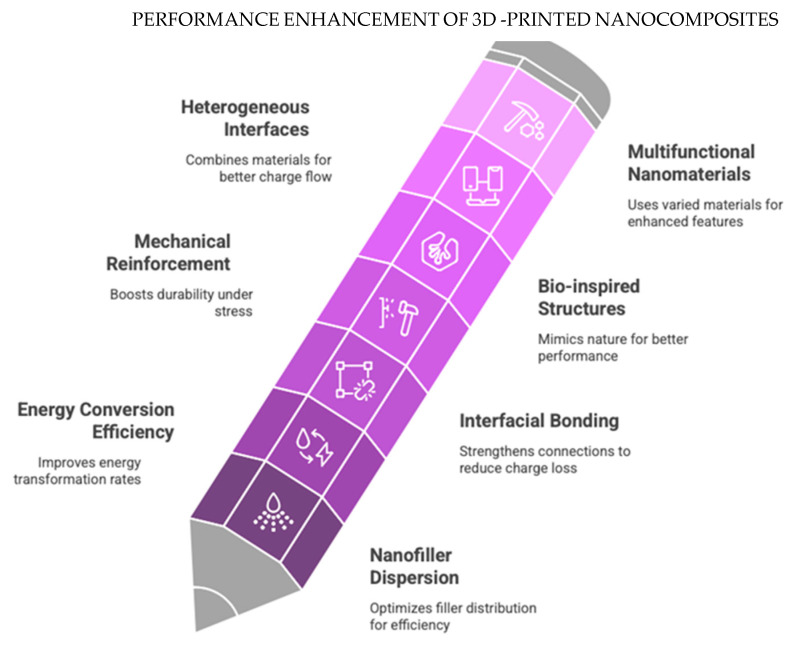
Performance enhancement.

Elements, like their material composition, notably shape the success of nanocomposites produced via 3D printing, how they are fabricated, and the treatments they undergo afterwards. Enhancing the spread of nanomaterials in the polymer matrix significantly improves mechanical strength, elevates electrical conductivity, and strengthens piezoelectric features. The surface functionalization of nanofillers, exemplified by MXene, graphene, and carbon nanotubes, significantly ameliorates interfacial adhesion, enhancing the energy conversion efficiency within nanogenerators. Furthermore, meticulously controlled printing parameters, such as the extrusion velocity, layer thickness, and curing techniques, are pivotal in reinforcing nanocomposites’ structural integrity and functional attributes. The overall view of the performance enhancement of 3D-printed nanocomposites is shown in Figure 15. Revolutionary practices, including employing various materials for printing and immediate curing actions, foster the development of exceptionally flexible and efficient energy-harvesting instruments. Table 4 shows a performance comparison of 3D-printed nanocomposites.

### 4.1. Mechanical Properties Enhancement

The mechanical properties of nanocomposites, encompassing tensile strength, elasticity, toughness, and durability, are markedly augmented owing to the reinforcing influence of nanofillers. The elevated aspect ratio and remarkable mechanical strength of nanofillers such as CNTs and graphene enhance the load-bearing capacity of the polymer matrix by efficiently transferring mechanical stress [112]. The robust interfacial interactions between the nanofiller and the polymer chain network mitigate microstructural deformations and amplify resistance to mechanical fatigue, rendering nanocomposites exceptionally resilient to recurrent mechanical deformations [130]. The integration of MXenes and metal oxide nanoparticles further bolsters mechanical stability by introducing supplementary hydrogen bonding and electrostatic interactions within the matrix [158]. This results in nanocomposites exhibiting a high strain tolerance, which is particularly advantageous for applications within flexible and wearable electronics. Lastly, nanocomposites based on nanocellulose present superb mechanical properties as a result of their rigid crystalline design and strong interfacial connections with polymer chains, thus making them appropriate for sustainable and compostable energy-harvesting systems [94]. The synergy between polymer matrices and nanofillers thus guarantees superior mechanical durability, enabling nanogenerators to endure prolonged mechanical stresses without a significant diminishment in performance.

### 4.2. Electrical Properties Enhancement

The electrical properties of nanocomposites, including electrical conductivity, charge storage capability, and dielectric permittivity, are significantly enhanced through the incorporation of conductive nanofillers. The addition of CNTs and graphene into polymer matrices creates percolation networks that facilitate efficient electron transport, thereby improving electrical conductivity and enhancing charge accumulation in nanogenerators [38]. The formation of conductive pathways ensures effective charge transfer, which is crucial for the efficient operation of piezoelectric and triboelectric nanogenerators [TENGs] [45]. MXenes, due to their layered structure and high metallic conductivity, further contribute to the enhancement of electrical properties in nanocomposites. Their large surface area and abundant functional groups enable strong interactions with polymer chains, leading to improved charge trapping and a higher output performance in energy-harvesting devices [92]. Additionally, metal oxide nanoparticles, such as ZnO and BaTiO_3_, contribute to the dielectric enhancement of polymeric matrices by increasing their permittivity, thereby amplifying the charge generation in nanogenerators [159]. The synergistic effect of multiple nanofillers, such as hybrid nanocomposites containing both CNTs and metal oxides, further enhances the electrical performance by combining high conductivity with strong dielectric properties. This multifunctional approach ensures that nanocomposites exhibit optimal electrical properties for energy-harvesting applications, enabling the development of high-output, flexible, and efficient nanogenerators.

### 4.3. Enhancement of Piezoelectric Properties

The piezoelectric characteristics of nanocomposites are markedly augmented through the synergistic interactions between polymeric matrices and piezoelectric nanofillers. For maximizing the energy conversion effectiveness of piezoelectric polymers, like polyvinylidene fluoride [PVDF], the alignment of dipoles is key [106]. The blend of nanofillers such as BaTiO_3_, ZnO, and MXenes with PVDF notably boosts its piezoelectric characteristics by assisting in the establishment of the electroactive β-phase, which boasts the most substantial piezoelectric coefficient [160]. By incorporating carbon nanotubes [CNTs] and graphene within piezoelectric nanocomposites, the mobility of charges and the transfer of stress are further improved, which elevates the total piezoelectric output [96]. These nanofillers possess conductive attributes that enhance the separation of charges during mechanical stress, which in turn elevates the voltage and current output in piezoelectric nanogenerators [PENGs] [38]. Furthermore, it has been documented that MXenes contribute to an improved dipole alignment and charge distribution in PVDF-based nanocomposites, thereby further elevating their piezoelectric performance [121].

By combining various nanofillers in hybrid nanocomposites, particularly within ZnO–MXene–PVDF frameworks, we observe a more substantial enhancement in piezoelectric characteristics, driven by the synergistic relationship among dielectric, conductive, and piezoelectric factors [130]. These hybrid technologies support the growth of next-level, high-performance energy-harvesting solutions that proficiently change mechanical energy into electrical energy, ensuring minimal energy loss.

### 4.4. Interface Engineering of 3D-Printed Nanogenerators

Interface engineering in three-dimensional-printed nanogenerators is fundamental in optimizing the dispersion and alignment of nanofillers, which profoundly impacts their energy conversion efficiency. Layer-by-layer additive manufacturing methodologies permit a meticulous regulation of the nanofiller distribution, thereby enhancing the uniformity of the composite matrix and guaranteeing optimal electrical and mechanical attributes [161]. The systematic deposition of functional nanomaterials, encompassing conductive nanofillers and polymeric matrices, yields well-defined interfacial bonding, consequently reducing charge loss and augmenting the charge transport efficiency in both piezoelectric and triboelectric nanogenerators [162]. Moreover, the deliberate engineering of interfacial interactions within multi-material three-dimensional-printed structures facilitates superior stress transfer and mechanical reinforcement, which are imperative for the durability and performance of nanogenerators when subjected to cyclic loading conditions [163].

Bio-inspired hierarchical nanostructures have emerged as a paradigm-shifting approach to augment the triboelectric and piezoelectric capabilities of 3D-printed nanogenerators. These architectural designs exploit multi-scale engineering principles to emulate naturally occurring energy-efficient formations, consequently enhancing the charge retention, energy accumulation, and mechanical flexibility [159]. For example, the amalgamation of 3D-printed WO3-UiO-66@reduced graphene oxide nanocomposites has exhibited an exceptional interfacial charge transfer, a critical factor for optimal energy conversion applications [164]. Moreover, the integration of heterogeneous interfaces, such as magnetoelectric coupling within composite materials, has been demonstrated to augment charge polarization and dielectric characteristics, thereby enhancing the energy harvesting efficacy [163]. The meticulous customization of these hierarchical configurations within 3D-printed nanogenerators facilitates the development of next-generation self-sustaining devices characterized by an improved stability, versatility, and operational efficiency.

The ongoing progression in interface engineering methodologies presents substantial prospects for the further enhancement of the operational efficacy of three-dimensional-printed nanogenerators. Novel techniques, featuring atomic-scale alterations of interfaces and the incorporation of multifunctional nanomaterials, demonstrate substantial potential for boosting thermoelectric, piezoelectric, and triboelectric attributes [164]. Furthermore, the synergistic amalgamation of diverse energy conversion mechanisms within a singular 3D-printed nanogenerator may facilitate the development of highly efficient and multifunctional energy-harvesting systems. Subsequent investigations ought to prioritize the refinement of printing techniques and the creation of novel nanocomposite formulations to extend the frontiers of interface engineering in the context of sustainable and high-performance nanogenerators.

### 4.5. Mechanisms of Materials That Enhance the Performance of 3D-Printed Nanogenerators

The integration of nanocomposite materials within three-dimensional-printed nanogenerators significantly enhances their energy harvesting efficacy by utilizing multiple mechanisms rooted in interfacial engineering, the modulation of charge transport, and coupling between mechanical and electrical properties. Central to this enhancement is the ability of nanocomposites to create heterogeneous interfaces that promote synergistic interactions between the active fillers and the polymer matrix, which would be otherwise unattainable in systems composed of a single component [162]. These carefully engineered interfaces facilitate effective stress transfer during mechanical deformation, consequently boosting the piezoelectric and triboelectric responses of the nanogenerator architectures [162]. For example, in nanocomposites incorporating MXenes, the large surface area and adjustable surface chemistry of the two-dimensional layers contribute to the efficient electron transfer and improved dielectric characteristics of the composite, thereby intensifying the triboelectric charge generation under cyclic loading conditions [161].

Another significant mechanism involves the formation of continuous conductive pathways within the nanocomposite. By integrating conductive nanofillers, such as carbon nanotubes (CNTs), silver nanowires (AgNWs), and reduced graphene oxide (rGO), into elastomeric or piezoelectric polymers, one can develop networks that effectively act as channels with a reduced resistance for charge collection and transport. This advanced conductive structure improves the electric flow and reduces dielectric energy losses, ultimately enhancing the overall conversion efficiency. Furthermore, the orientation of these nanofillers during the 3D printing process, whether through extrusion or magnetic field-assisted methodologies, can induce anisotropic characteristics in electrical and mechanical properties, further optimizing the directional energy output [164].

Interfacial polarization represents another crucial element that contributes to enhancing performance. The combination of nanofillers with increased dielectric constants in a lower permittivity matrix results in boundary layer polarization, which boosts the dielectric constant of the composite as a whole. This dielectric enhancement increases the energy storage capacity and strengthens the triboelectric potential, which is vital for the functionality of triboelectric nanogenerators. In piezoelectric nanocomposites, nanofillers act as nucleation agents, promoting the crystallization of β-phase structures found in PVDF-based matrices, known for their extraordinary piezoelectric properties. The ceramic nanoparticles incorporated in BaTiO_3_-PVDF composites not only produce a significant piezoelectric response but also help enhance the matrix’s crystallinity, leading to an improved electromechanical conversion efficiency [161].

Moreover, the surface functionalization of nanofillers is strategically employed to enhance the interfacial compatibility with the polymer matrix and to introduce active sites conducive to charge trapping or dipole alignment [162]. Functional groups, including –OH, –COOH, or amine groups, located on the nanofiller surface create hydrogen bonds or covalent ties with the polymer matrix, thus preventing the clumping of fillers and facilitating an even dispersion. This uniformity is critical in maintaining the consistent performance of the nanogenerators over repeated operational cycles, a factor that is essential for ensuring long-term durability [159].

## 5. Advantages of 3D-Printed Nanocomposites in Nanogenerators

### 5.1. Customization and Complex Geometries

A salient advantage of three-dimensional [3D] printing technology in the context of nanogenerators lies in its capacity to produce highly intricate and tailored geometrical configurations that are challenging, if not unfeasible, to realize through conventional manufacturing processes [165]. The methodology of layer-by-layer fabrication facilitates meticulous regulation throughout the internal architecture, thereby permitting the design of optimized surface morphologies that significantly augment the charge generation and enhance the energy harvesting efficacy [166]. Furthermore, the implementation of Digital Light Processing [DLP] 3D printing offers superior resolution and precision, which are imperative for the fabrication of micro- and nanoscale structures intended for energy harvesting [167]. This proficiency is especially advantageous for wearable and implantable nanogenerators that necessitate conformal geometries to seamlessly integrate with the movements of the human body [168].

### 5.2. Material Versatility and Functionalization

The domain of 3D printing provides remarkable versatility in the selection of materials, thereby facilitating the incorporation of various functional nanocomposites to augment the attributes of nanogenerators. Energy-harvesting applications frequently utilize piezoelectric and triboelectric polymers, particularly polyvinylidene fluoride [PVDF] and its copolymers, due to their remarkable ability to convert electromechanical energy efficiently [149]. The introduction of nanoparticles such as ZnO, BaTiO3, and MXene into 3D-printed polymer matrices further amplifies the energy-harvesting functionalities by enhancing the charge mobility and bolstering mechanical resilience [169,170]. Moreover, the advent of multi-material 3D printing facilitates the integration of conductive electrodes within the nanogenerator structure, thereby obviating the necessity for supplementary assembly processes and enhancing the overall device performance [171].

### 5.3. Scalability and Cost-Effectiveness

In comparison to traditional fabrication methodologies, 3D printing presents a financially viable and scalable paradigm for the production of nanogenerators [172]. The additive nature of 3D printing minimizes material wastage, rendering it a sustainable and economically favorable alternative for large-scale manufacturing [173]. Additionally, the capability to fabricate fully functional nanogenerators in a singular production step reduces the intricacy of the device assembly, culminating in decreased labor and manufacturing expenses [174]. Progressions in high-throughput 3D printing technologies, such as multi-jet fusion and stereolithography, further bolster scalability by facilitating the mass production of energy-harvesting devices characterized by consistent quality and performance metrics [175].

### 5.4. Enhanced Mechanical and Environmental Adaptability

3D-printed nanocomposites enable the creation of flexible and stretchable nanogenerators that possess the capacity to adapt to fluctuating environmental parameters [176]. The ability to print soft, deformable materials permits the engineering of energy-harvesting devices that can endure repetitive bending, stretching, and mechanical impacts, thus rendering them suitable for applications including wearable sensors and smart textiles [82]. Alongside this, progress in self-restorative and green nanocomposites bolsters the lasting sustainability and toughness of these devices [177].

### 5.5. Biocompatibility and Clinical Integration

A significant plus of 3D-printed nanogenerators, especially in biomedical and wearable applications, is the potential to weave biocompatible, safe, and regulation-approved materials into elaborate energy-harvesting designs. In contrast to conventional fabrication techniques that frequently depend on rigid or potentially cytotoxic materials, additive manufacturing facilitates the meticulous formulation of nanocomposites specifically designed for biological compatibility. The use of polyvinylidene fluoride (PVDF) alongside its copolymer P(VDF-TrFE) is prevalent in flexible bioelectronics, which is attributed to their beneficial piezoelectric qualities and lower levels of cytotoxicity [178]. Empirical studies have validated their compatibility with mammalian cells, rendering them appropriate for implantable biosensors, neural stimulators, and pacemakers. Similarly, substances like Ecoflex and polydimethylsiloxane (PDMS) are largely adopted in devices that interact with skin and are implantable, thanks to their non-reactive characteristics, adaptability, and FDA endorsement for biomedical usage [179]. Their ability to conform to soft tissues without provoking inflammation or degradation positions them as optimal substrates for self-powered healthcare systems.

Furthermore, utilizing naturally sourced biomaterials, namely silk fibroin and chitosan, yields specific ecological and health benefits. These products can decompose naturally, have qualities that combat bacteria, and reveal an outstanding compatibility with living tissue, which helps to reduce fears regarding extended implantation or persistent buildup. For example, chitosan has been awarded an FDA recognition for its roles in wound dressings, tissue engineering supports, and controlled medication delivery, thereby corroborating its safety standing for the evolution of nanogenerators [180,181]. The capacity to amalgamate such bio-originated materials with functional nanofillers (e.g., BaTiO_3_, CNTs, or MXenes) further augments their electrical output while preserving biological safety.

From a translational perspective, the adaptability of 3D printing enables the creation of patient-specific designs that mitigate surgical complications and enhance device–tissue interfacing. Moreover, materials developed through additive manufacturing can be assessed in accordance with internationally recognized biological evaluation standards, thereby expediting their pathway toward clinical implementation [182]. Therefore, the interaction between 3D printing techniques and biocompatible compounds not only boosts the performance of nanogenerators but also assures their safe deployment in real-life biomedical scenarios, representing key progress toward forthcoming self-operating healthcare innovations.

## 6. Challenges of 3D-Printed Nanocomposites in Nanogenerators

### 6.1. Material Limitations and Printability

Despite the substantial progress made in 3D printing technology, the printability of functional nanocomposites remains a significant challenge [167]. The rheological properties, viscosity, and curing dynamics of nanocomposite inks greatly affect the resolution and structural integrity of the produced nanogenerators [183]. Achieving a uniform distribution of nanoparticles within polymer matrices is essential to ensure uniform mechanical and electrical properties; however, issues such as nanoparticle clumping and phase separation often arise during the printing process [170]. Furthermore, some piezoelectric and triboelectric materials show brittleness, which can lead to the emergence of cracks and defects during the additive manufacturing process [184].

### 6.2. Mechanical and Environmental Stability

The enduring stability of 3D-printed nanogenerators represents a critical issue, particularly for applications that require continuous mechanical deformation or exposure to extreme environmental conditions [149]. Fluctuations in temperature, humidity, and mechanical stress can compromise the performance of nanogenerators by negatively impacting the charge retention and material integrity [174]. To address these challenges, researchers are exploring methods such as encapsulation with protective coatings and the use of highly flexible and resilient polymer matrices [80]. Furthermore, advancements in self-healing and stretchable materials show promise for enhancing the mechanical durability of 3D-printed nanogenerators intended for wearable and flexible electronic applications [82].

### 6.3. Energy Conversion Efficiency and Performance Optimization

Although 3D printing enables the creation of innovative designs for nanogenerators, achieving high energy conversion efficiency remains a significant challenge [92]. The effectiveness of piezoelectric and triboelectric nanogenerators depends on various factors, including the electrode orientation, charge trapping methods, and interface adhesion [185]. Researchers are exploring novel methodologies, such as auxetic structure-assisted energy-harvesting and hierarchical nano-architectures, to enhance the energy output and mechanical strength [186]. Furthermore, investigations into hybrid nanogenerators that combine multiple energy capture methods, like piezoelectric–triboelectric and pyroelectric–thermoelectric combinations, are actively underway to boost the overall energy efficiency [93,187]. Table 5 shows the advantages and challenges of 3D-printed nanocomposites in nanogenerators. Table 6 compares the properties of nanocomposites that are 3D-printed versus those made using conventional methods

## 7. Three-Dimensional Printing in Flexible Nanogenerators

Three-dimensional [3D] printing has emerged as a revolutionary method for fabricating flexible nanogenerators, allowing precise control over the material composition, structural design, and mechanical properties. Additive manufacturing enables the integration of functional nanomaterials, such as MXene, graphene, and piezoelectric ceramics, into polymer matrices, thereby enhancing energy conversion efficiency. Direct ink writing [DIW], fused deposition modeling [FDM], extrusion-based 3D printing, robocasting, and stereolithography [SLA] are prominent techniques used to create customized, flexible, and stretchable nanogenerator architectures. These advancements lead to the development of lightweight, wearable, and self-sustaining energy devices with potential applications in biomedical sensing, electronic skin, and smart textile solutions.

### 7.1. Three-Dimensional Printing Technologies Utilized in Nanogenerators

**Figure 16:**
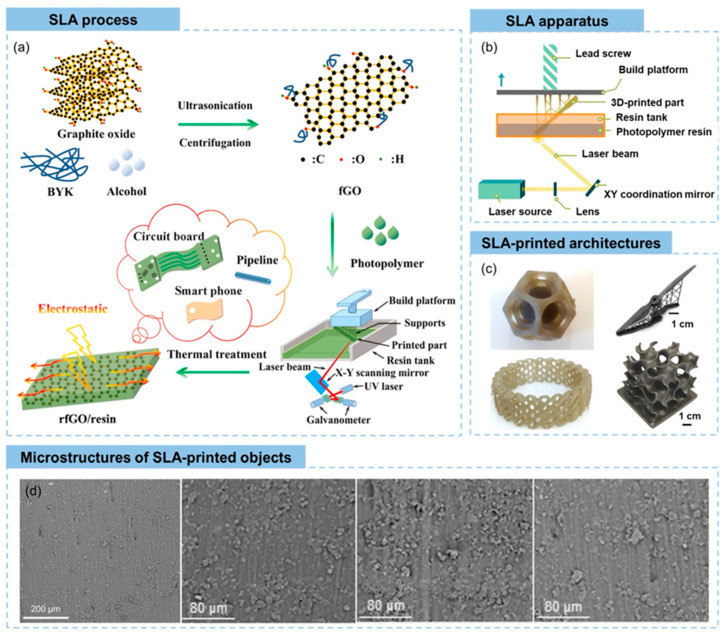
(**a**) A conventional manufacturing methodology for the synthesis of functionalized reduced graphene oxide/resin composites through stereolithography [SLA]. (**b**) A schematic representation of the SLA apparatus. (**c**) Commonly produced graphene/polymer composite structures utilizing SLA and (**d**) microscopic morphological characteristics of graphene nanocomposites fabricated via SLA [86].

**Figure 17:**
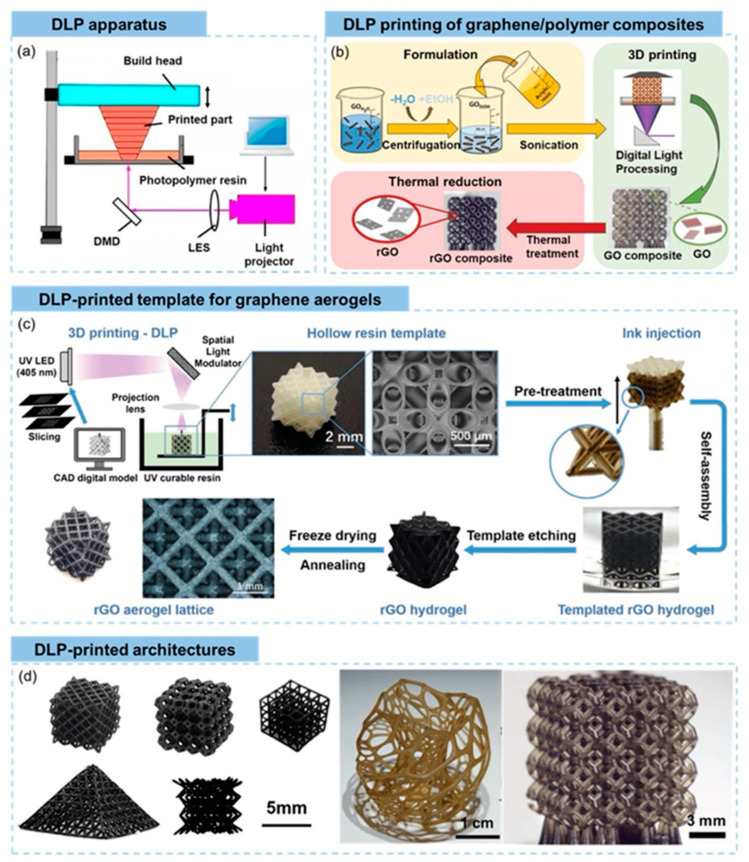
(**a**) A schematic representation of the Digital Light Processing [DLP] apparatus. (**b**) A standard fabrication methodology for graphene/polymer composites utilizing DLP printing techniques. (**c**) The fabrication methodology of graphene aerogel lattices derived from DLP-printed hollow structures. (**d**) Conventional architectures of graphene and graphene/polymer composites produced through DLP printing [86].

Three-dimensional printing technologies have emerged as pivotal methodologies in the fabrication of flexible nanogenerators, which can be attributed to their capacity to construct intricate geometries with remarkable precision and material adaptability. In the spectrum of popular techniques, stereolithography [SLA] and Digital Light Processing [DLP] are especially distinguished for their excellent resolution potential and are widely employed in the generation of micro-structured products that are important for effective energy harvesting [165,166,167,168]. Both SLA and DLP leverage the photopolymerization of resin materials, facilitating the formation of finely detailed features that are essential for augmenting the surface charge density in triboelectric and piezoelectric nanogenerators [149,174]. Figure 16 and Figure 17 show the process of SLA and DLP, respectively. Recent investigations have demonstrated the successful application of DLP in the fabrication of environmentally sustainable nanocellulose-based films utilized in flexible triboelectric nanogenerators [176].

**Figure 18:**
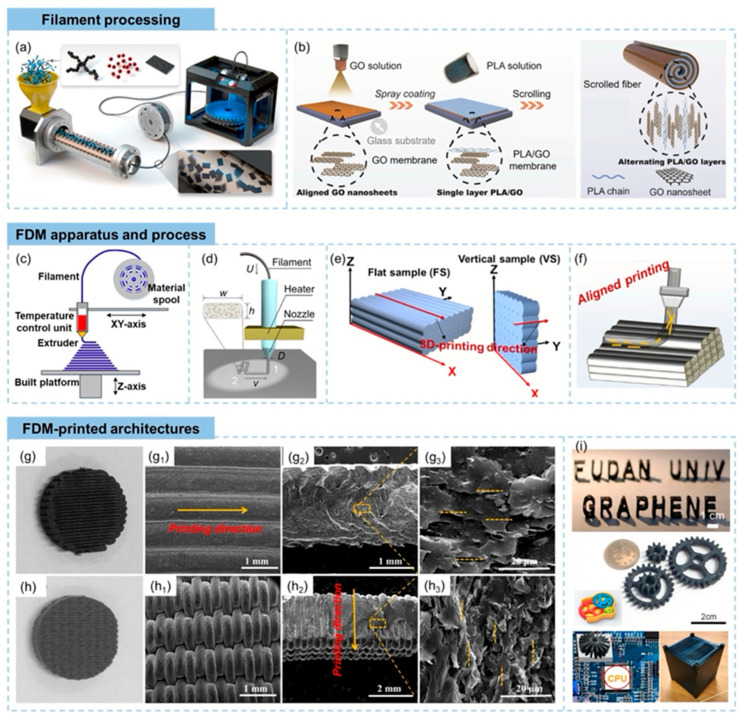
The FDM technique for 3D printing. Filament processing: (**a**) the preparation of graphene/polymer filaments via the screw-extrusion method and (**b**) a schematic diagram of the scrolled fiber with highly aligned GO for FDM. The FDM apparatus and process: (**c**) a schematic illustration of FDM, (**d**) the typical FDM process for graphene/polymer composites, which includes (1) an extrusion and (2) a deposition process, and (**e**,**f**) the aligned printing of flat and vertical samples. FDM-printed graphene/polymer composite architectures: (**g**,**h**): Exhibits a representative specimen printed with a designated orientation or pattern, while the subpanels (**g_1_**–**g_3_**) provide comprehensive SEM imagery at varying magnifications. The SEM images elucidate the stratified architecture, displaying the orderly arrangement of printed layers and the internal microstructure, which presumably accentuates the directional characteristics of the printing process. (h): Illustrates an alternative printed specimen employing a distinct orientation or fabrication strategy. Subpanels (**h_1_**–**h_3_**) showcase SEM images that reveal the unique microstructural configurations, indicating discrepancies in layer stacking and potential variations in porosity or interlayer adhesion. (**i**) macroscopic photos [86].

Recognized for its simplicity and economic efficiency, fused deposition modeling [FDM] is a prominent method in 3D printing, supporting various thermoplastic materials, like conductive and adaptable polymers [189,190]. Figure 18 shows the process of FDM. The deposition mechanism of FDM, which operates layer-by-layer, enables the addition of conductive filaments, such as carbon-based composites and blends of PVDF, often used in devices for piezoelectric energy harvesting [169,187]. Its scalability and material versatility render it suitable for prototyping wearable nanogenerators where mechanical flexibility is of paramount importance [175,189].

**Figure 19:**
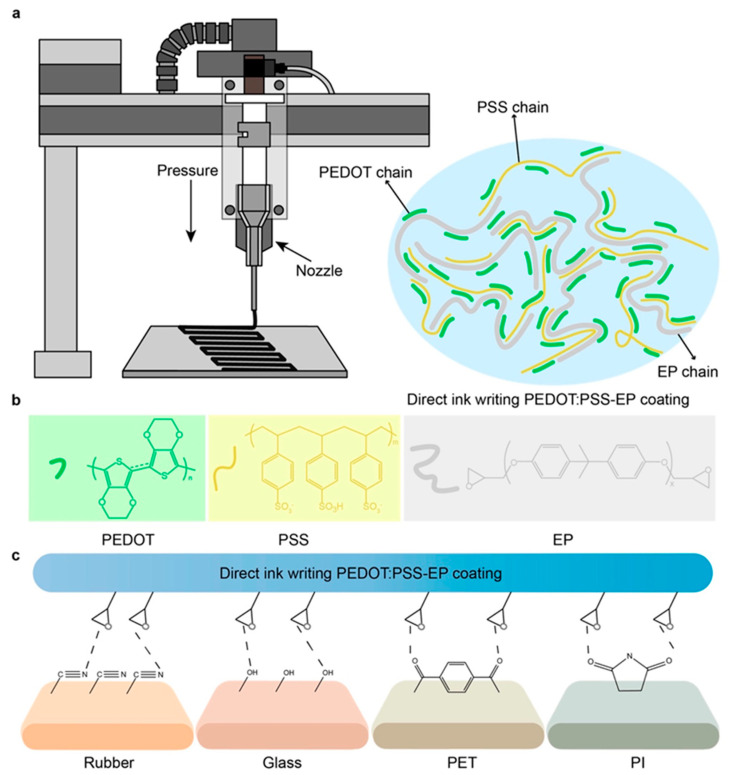
The design of PEDOT: the PSS-EP coating utilizing direct ink writing [DIW]. (**a**) The mechanisms underlying DIW and the thermal curing of the PEDOT: the PSS-EP coating. (**b**) The compositional framework of PEDOT: the PSS-EP coating, encompassing both PEDOT: PSS and EP. (**c**) Potential adhesion mechanisms of PEDOT: PSS-EP coatings in relation to various substrates [89].

A frequently adopted technique is direct ink writing [DIW], which facilitates the printing of viscoelastic inks infused with functional fillers, including BaTiO_3_, ZnO nanoparticles, and carbon nanomaterials [170,183]. Figure 19 shows the process of DIW. DIW proves advantageous for the fabrication of flexible piezocomposites with a meticulously controlled filler alignment, thereby enhancing the anisotropic characteristics of nanogenerators and improving their energy conversion efficacy [183]. Notably, DIW has played a crucial role in integrating bio-based materials, such as lignin and cellulose derivatives, to engineer sustainable and biodegradable nanogenerators [191].

Inkjet printing serves as a distinct additive manufacturing approach that has been applied for layering thin, consistent coatings of conductive inks and functional nanomaterials on adaptable surfaces [170,192]. Its non-contact nature and elevated patterning resolution render it particularly suitable fofabricatingof micro-scale electrodes and interconnects within flexible electronic systems [192,193]. Recent advancements have amalgamated inkjet printing with soft materials to engineer multifunctional energy-harvesting components integrated with sensors and supercapacitors [171,174].

Innovative techniques like Laser-Induced Graphene [LIG] printing and multi-material hybrid printing have significantly expanded the design options available in the creation of nanogenerators [191,192]. LIG facilitates the direct conversion of polymeric substrates into conductive graphene structures, thereby allowing for the seamless integration of electrodes within flexible devices [159]. Hybrid approaches, which amalgamate DLP, FDM, and DIW techniques, have enabled the simultaneous deposition of structural, conductive, and piezoelectric materials within a singular process, thereby paving the path for highly integrated and compact energy devices [194,195].

Furthermore, contemporary research has elucidated the application of three-dimensional [3D] printing techniques for the fabrication of bi-stable asymmetric raceway configurations and architecturally designed β-PVDF reservoirs, thereby augmenting the mechanical and piezoelectric efficacy of flexible nanogenerators [169,196]. The versatility inherent in 3D printing further facilitates the meticulous integration of non-toxic and environmentally sustainable materials, such as Ga-doped ZnO and PEDOT: PSS thermoelements, rendering it exceptionally appropriate for the development of wearable and sustainable energy applications [80,197].

The diverse spectrum of three-dimensional printing techniques—covering advanced photopolymerization methods like stereolithography [SLA] and Digital Light Processing [DLP], alongside adaptable strategies such as fused deposition modeling [FDM], direct ink writing [DIW], inkjet printing, and intricate hybrid systems—has driven significant advancements in the structural development, material synthesis, and performance improvement of flexible nanogenerators [165,172,198]. Table 7 provides the analysis of additive manufacturing techniques.

### 7.2. Advantages of 3D Printing in Fabricating Flexible Energy-Harvesting Devices

Three-dimensional printing technologies provide an unprecedented design freedom, enabling the creation of intricate, customized architectures that significantly enhance the efficiency of flexible nanogenerators [165,170]. Unlike traditional manufacturing methods, 3D printing offers detailed control over geometric features, such as surface smoothness, porosity, and internal lattice configurations, which are crucial for maximizing the surface area and improving triboelectric or piezoelectric phenomena [149,183,189]. For example, triboelectric nanogenerators created using Digital Light Processing [DLP] and stereolithography [SLA] benefit from micro-scale surface texturing that enhances the charge separation and increases the energy generation [166,174].

Additionally, a significant advantage is recognized in the capacity to utilize various materials—such as flexible polymers, conductive inks, ceramics, and bio-sourced composites—thereby aiding the advancement of multifunctional energy devices [191]. Techniques like direct ink writing [DIW] and fused deposition modeling [FDM] facilitate the integration of nanomaterials, namely BaTiO_3_, ZnO, PVDF, and graphene, within adaptable matrices, ensuring mechanical flexibility and electrical efficiency [170,191]. Furthermore, multi-material 3D printing promotes the seamless amalgamation of electrodes, energy storage components, and sensing layers into a single platform, leading to compact, lightweight, and wearable energy systems [174,194].

The realm of three-dimensional printing offers remarkable benefits for rapid prototyping and scalability. It enables swift design iterations, thereby reducing development timelines and associated costs, which is particularly advantageous in wearable and biomedical contexts where device customization is essential [165,190]. Furthermore, the commitment to sustainable practices, including the use of lignin, nanocellulose, and inks containing Ga-doped ZnO, aligns with the increasing demand for eco-friendly and biodegradable energy systems [197]. The additive nature of 3D printing significantly reduces material waste compared to subtractive methods, contributing to sustainable manufacturing practices [198].

### 7.3. Challenges in Integrating 3D-Printed Structures with Flexible Substrates

Despite the significant benefits, many challenges hinder the seamless integration of 3D-printed nanogenerators with flexible substrates. A primary concern involves the differences in mechanical properties between rigid 3D-printed materials and adaptable substrates, which can lead to delamination, fracturing, or mechanical failure during repeated bending or stretching [199]. Materials like ceramics and thermoplastics used in SLA, DLP, or FDM often exhibit a high stiffness, thereby limiting their compatibility with elastomeric substrates [167,175].

Achieving strong adhesion at the interface between the printed layers and flexible films poses an additional challenge, especially in multi-material and hybrid printing methods [192,193]. Differences in thermal expansion coefficients and surface energies can lead to insufficient bonding, negatively affecting the durability of the device [80]. Strategies like surface treatment, plasma activation, or the use of intermediate adhesive layers are often required to improve the interfacial stability, though this might increase the processing complexity [192].

A further limitation relates to the printability and rheological characteristics of functional inks. Direct ink writing [DIW] and inkjet printing require inks with a precisely calibrated viscosity, surface tension, and particle dispersion to prevent nozzle blockage and ensure uniform deposition. This task becomes especially complex when integrating nanomaterials or bio-based additives [170,191]. Additionally, the subsequent processing steps, such as curing, sintering, or annealing, may affect the mechanical flexibility and operational performance of the printed structures [172,175].

Miniaturization and resolution constraints pose significant challenges. While SLA and DLP provide high resolution, scaling production for large-area flexible devices without compromising structural integrity remains a complex challenge [166,168]. In contrast, the relatively low resolution of FDM limits its applicability in micro-scale energy devices, where intricate patterns are crucial for optimizing the output performance [189,190].

Integrating energy-harvesting devices with flexible circuitry and storage solutions, such as supercapacitors and micro-batteries, necessitates the joint printing of multiple functional layers that exhibit diverse electrical, mechanical, and chemical properties. This remains a significant technical challenge [170,171,194]. Addressing these integration challenges requires further investigation into material formulation, interfacial engineering, and process optimization [192,199].

## 8. Recent Advances in 3D-Printed Nanocomposite Nanogenerators

### 8.1. Three-Dimensional-Printed Piezoelectric Nanogenerators [PENGs]

**Figure 20:**
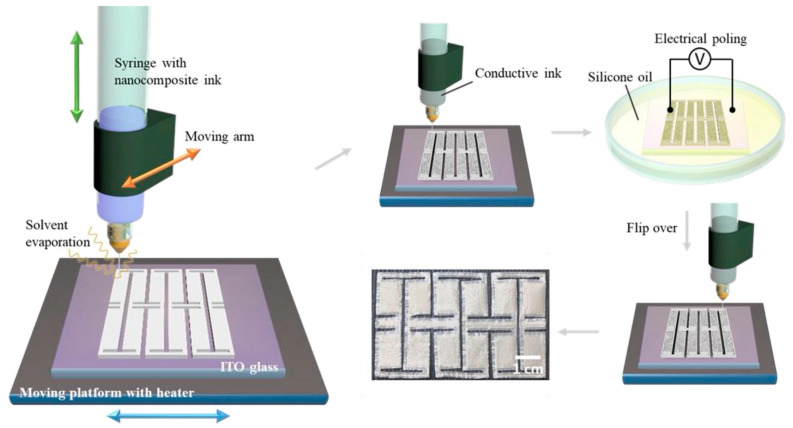
A schematic representation illustrating the methodology employed in the fabrication process of the three-dimensional-printed piezoelectric nanogenerator [PENG] [90].

Piezoelectric nanogenerators [PENGs] have made significant progress thanks to advances in three-dimensional printing techniques, which enable meticulous structural manipulation and an enhanced operational efficiency. Figure 20 shows the schematic representation of the fabrication process for 3D-printed PENGs. Recent studies have demonstrated the effectiveness of incorporating nanomaterials such as MXenes, graphene derivatives, and doped ZnO into printable piezoelectric polymers to boost energy harvesting efficiency [184]. The strategic arrangement of graphitic carbon nitride nanosheets within 3D-printed PENGs has been shown to greatly enhance the piezoelectric response by optimizing the orientation of dipoles [185].

Novel configurations have been developed, including auxetic structures that enhance mechanical adaptability while maximizing the energy conversion efficiency of printed nanogenerators [92]. The combination of electrostrictive salt cocrystals with polymer matrices has resulted in a superior output performance, underscoring the importance of advanced material design in the field of 3D-printed PENGs [96]. Furthermore, the inclusion of Ga-doped ZnO in flexible PENGs has broadened their applicability in wearable energy-harvesting frameworks [80].

Multilayer architectures in 3D-printed PENGs are vital for optimizing the charge generation and mechanical resilience. An investigation into metamaterial-based PVDF nanogenerators revealed how programmable layer structuring significantly enhances the power output, making them especially suitable for electronic skin applications [82]. Furthermore, the development of hybrid polymer composites, such as PVDF-TrFE/MXene blends, has resulted in improved energy conversion properties due to optimized layer-by-layer printing methodologies [128].

### 8.2. Three-Dimensional-Printed Triboelectric Nanogenerators [TENGs]

**Figure 21:**
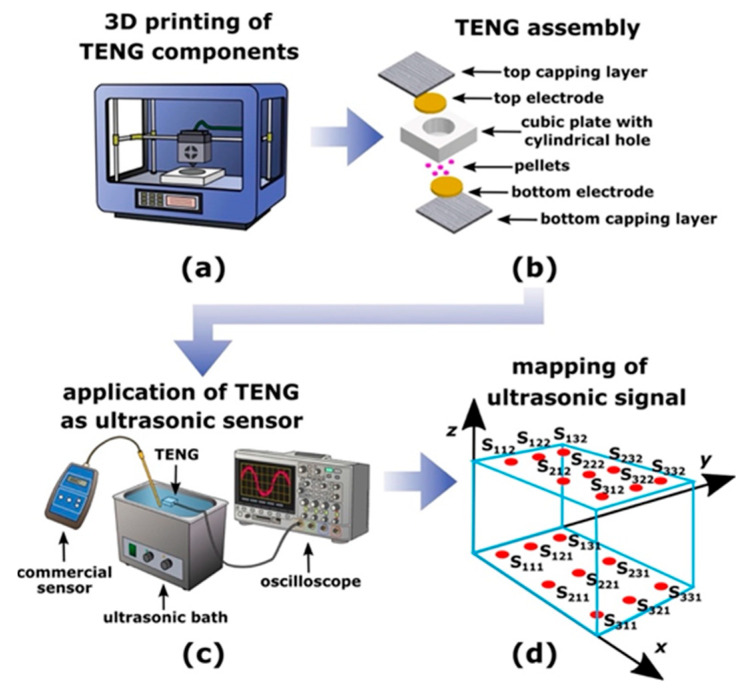
(**a**) A schematic illustration of the components of the triboelectric nanogenerator [TENG]. (**b**) The configuration of their assembly, (**c**) the utilization of the TENG for the detection of ultrasonic waves, (**d**) and the assessment of the acoustic power distribution within an ultrasonic bath [200].

Enhancements in triboelectric nanogenerators [TENGs] have been largely driven by the adoption of 3D printing methods, which create intricate microstructures that increase the surface charge density and enhance triboelectric interactions. A schematic representation of the 3D printing process for TENG components is shown in Figure 21. Researchers have investigated innovative polymer formulations specifically designed for 3D-printed TENGs, resulting in significant improvements in charge generation and mechanical resilience [186]. The use of laser-patterned surfaces within TENGs has been empirically demonstrated to substantially improve the energy output by increasing the efficiency of the contact electrification [149].

A particularly promising advancement in the realm of 3D-printed TENGs is the emergence of multi-material printing methodologies that facilitate the precise integration of triboelectric pairs within a single device [174]. The engineering of serrated contact architectures has further optimized the charge separation and energy harvesting efficiency, as demonstrated in practical applications such as wearable motion sensors [201]. Moreover, the incorporation of textile-based TENGs through 3D printing has unveiled new possibilities for flexible and portable energy-harvesting solutions [202].

The optimization of layer structuring in 3D-printed TENGs has been strategically developed to achieve exceptional output characteristics. The advent of in situ foam 3D printing has facilitated the production of lightweight, flexible TENGs with enhanced charge storage capabilities, making them highly suitable for self-powered sensing applications [203]. Furthermore, employing FDM-driven printing approaches to fabricate CNT-ZnO core–shell nanostructures has led to significant advancements in triboelectric properties by leveraging interactions provided by a large surface area [177].

### 8.3. Three-Dimensional-Printed Hybrid Nanogenerator

Hybrid nanogenerators that combine both piezoelectric and triboelectric mechanisms have attracted significant scholarly attention due to their capability to simultaneously harvest energy from various mechanical stimuli. The emergence of 3D printing technology has been crucial in the development of hybrid nanogenerators, employing custom material compositions and structural designs to enhance the effectiveness of energy conversion [204]. The use of multi-material voxel-based printing has enabled the effective integration of piezoelectric and triboelectric components within a single device, thereby improving energy-harvesting capabilities [39].

A notable innovation in hybrid nanogenerators is their implementation in real-time monitoring systems. A 3D-printed stretchable hybrid nanogenerator has been skillfully utilized in smart tires to enable onboard tread wear monitoring, thereby demonstrating the practical potential of these advanced systems [205]. The development of cellulose nanocrystal-based pyro–piezoelectric hybrid nanogenerators has further expanded the operational scope of self-powered healthcare monitoring devices [93].

Significant advancements have also emerged in the design of hybrid nanogenerators. The integration of bidirectional rotary structures has enabled the efficient harvesting of mechanical energy from various orientations, making them particularly suitable for self-powered electronic applications [204]. Additionally, the incorporation of zinc-ion hybrid capacitors with nanogenerators has facilitated the development of all-in-one self-powered energy wristbands [187]. Recent studies on the fabrication of hybrid nanogenerators for wearable applications emphasize the need to optimize the material selection and layer architecture to enhance device longevity and the operational performance [116].

The convergence of 3D printing technology and nanogenerator innovation has led to transformative advancements in energy harvesting. The ability to engineer customized nanocomposite structures with enhanced piezoelectric, triboelectric, and hybrid properties has opened pathways for developing next-generation self-powered systems. Future research efforts should focus on refining 3D printing methodologies to further enhance the efficiency, durability, and practical integration of nanogenerators into real-world applications [17].

In recent years, the combination of three-dimensional [3D] printing technologies with nanocomposite materials has resulted in significant advancements in the development of nanogenerators. These devices, which include piezoelectric, triboelectric, and hybrid nanogenerators, have shown improved performance metrics and innovative designs.

## 9. Comparative Analysis of 3D-Printed Nanogenerators

### 9.1. Performance Metrics: Voltage, Power Density, and Efficiency

The performance of nanogenerators created through 3D printing is typically assessed using key metrics such as the voltage output, energy density, and energy conversion efficiency. Understanding these indicators is crucial for evaluating the practicality of nanogenerators in real-world energy-collection scenarios. A significant advantage of employing 3D printing techniques in the production of nanogenerators is the precise control over both the material composition and structural design, which enhances triboelectric and piezoelectric properties [206].

For example, triboelectric nanogenerators [TENGs] created using optimized buoyancy-gravity conductive 3D printing techniques have demonstrated significantly improved voltage outputs, surpassing 250 V. This makes them suitable for large-scale wave energy-harvesting projects [206]. Similarly, 3D-printed multi-material smart flooring tiles that integrate triboelectric energy harvesters have achieved power densities of up to 2.5 W/m^2^, which is sufficient for enabling real-time security surveillance and self-sustaining sensing applications [207]. Furthermore, a study focused on the direct 3D printing of triboactive polymer layers onto stretchable conductive fabrics has reported a 30% increase in energy conversion efficiency compared to conventional TENG fabrication methods, significantly enhancing the overall effectiveness of wearable energy devices [208].

Complementing TENGs, piezoelectric nanogenerators [PENGs] have also harnessed the potential of 3D printing approaches that enable the accurate electrical poling of polyvinylidene fluoride [PVDF] designs. Scientific investigations have shown that the combination of additive manufacturing with electrical poling significantly intensifies the piezoelectric response of PVDF-based nanogenerators, consequently enhancing the power output and efficiency [209]. The incorporation of reconfigurable 3D-printed metasurfaces has further demonstrated the ability to focus acoustic energy with a high efficiency, thereby improving the energy harvesting potential at low frequencies [210]. These technological advancements highlight how 3D printing facilitates the creation of nanogenerators with customized performance attributes tailored to meet specific application requirements.

### 9.2. Material and Processing Cost Analysis

The financial viability of 3D-printed nanogenerators is significantly influenced by both the materials used and the complexity of the manufacturing techniques employed. Standard production techniques for triboelectric nanogenerators [TENGs] and piezoelectric nanogenerators [PENGs] often require expensive materials, such as rare-earth metals, or intricate lithographic processes, thus limiting scalability [211]. In contrast, recent advancements in 3D printing methods have enabled the use of low-cost, biodegradable materials and conductive inks, resulting in a substantial reduction in material costs [212]. For example, nanogenerators produced through dual-head additive manufacturing using bio-derived materials have demonstrated performance levels comparable to traditional models while also enhancing sustainability and cost efficiency [212].

The application of direct Digital Light Processing [DLP] in the 3D printing of polymeric nanogenerators has optimized the fabrication process, enabling expedited prototyping with minimal waste generation and reduced processing costs [213]. Moreover, utilizing plasma jet printing for the deposition of silver nanoparticle [AgNP] electrodes onto fabric-based TENGs offers a cost-effective alternative to vacuum deposition techniques, achieving a higher conductivity at significantly lower costs [211]. These advancements highlight the financial benefits of incorporating 3D printing methodologies for the scalable and economically viable production of nanogenerators.

### 9.3. Mechanical Durability—Flexibility and Biocompatibility

The mechanical strength, adaptability, and compatibility with biological systems are crucial factors influencing the long-term viability of 3D-printed nanogenerators, especially in the fields of wearable technology, healthcare solutions, and flexible energy-collection systems. The inherent flexibility of 3D-printed materials, along with the ability to precisely adjust structural designs, has led to significant improvements in the mechanical resilience of nanogenerators [208].

Empirical investigations have shown that 3D-printed nanogenerators incorporating stretchable conductive fabrics and triboactive polymer layers can withstand cyclic mechanical deformations without a significant performance degradation. For instance, a TENG produced via 3D printing on stretchable conductive materials maintained a reliable energy output after 10,000 bending cycles, highlighting its outstanding durability for wearable applications [208]. Similarly, a flexible, multi-material TENG designed for self-powered force sensing exhibited a consistent energy conversion efficiency under repeated compression and stretching, emphasizing its resilience in dynamic environments [214].

The integration of sustainable and compostable materials in 3D-printed nanogenerators has garnered considerable academic attention regarding biocompatibility. A significant advancement in this area is the development of TENGs using 3D-printed nanocellulose films, which have non-toxic and biodegradable properties, making them especially suitable for monitoring human motion and biomedical applications [176]. Furthermore, polylactic acid [PLA]-based TENGs have been explored as an eco-friendly alternative to conventional synthetic polymers, demonstrating compatibility with biological systems and effective energy-harvesting capabilities [15].

Another crucial aspect of mechanical performance concerns the durability of 3D-printed nanogenerators when subjected to environmental stressors. A recent scholarly study examined the stability of reconfigurable 3D-printed metasurfaces designed for low-frequency acoustic energy harvesting, revealing that the devices maintained their structural integrity and functional efficacy despite changes in temperature and humidity [210]. This research shows that 3D printing not only enhances the mechanical integrity of nanogenerators but also promotes the development of dynamic and adjustable energy-harvesting systems that can perform well in various environmental conditions.

The advancements in 3D printing innovation have significantly enhanced the mechanical robustness, versatility, and biocompatibility of nanogenerators, increasing their applicability in real-life scenarios. The ability to create mechanically robust and environmentally friendly energy-harvesting devices through additive manufacturing is paving the way for the next generation of self-powered wearable and biomedical technologies.

Table 8 showcases the comparison of 3D-printed and conventional nanogenerators.

## 10. Current Challenges in 3D-Printed Nanogenerators

### 10.1. Material Selection and Ink Formulation Challenges

The efficacy of three-dimensional-printed nanogenerators [NGs] is heavily dependent on the careful selection of suitable materials and the precise formulation of printable inks. Unlike traditional fabrication methods, additive manufacturing requires materials that not only show favorable electrical and mechanical properties but also possess appropriate rheological characteristics for processes like extrusion or photopolymerization [174]. Compounds such as polytetrafluoroethylene [PTFE], polydimethylsiloxane [PDMS], and fluorinated ethylene propylene [FEP] are frequently used in triboelectric applications due to their excellent charge generation capabilities. However, these materials often lack compatibility with most 3D printing techniques, mainly because of their low adhesion, high viscosity, or lack of photopolymerization ability [166].

In piezoelectric nanogenerators, materials such as polymer–ceramic composites derived from polyvinylidene fluoride and its variants are commonly used, providing a blend of flexibility and enhanced piezoelectric performance. However, achieving an even distribution of piezoelectric components, like barium titanate [BaTiO_3_] or zinc oxide [ZnO], within the polymer matrix poses significant challenges, which is linked to issues such as particle clustering, phase separation, and sedimentation during the printing process [183]. These challenges greatly affect the mechanical integrity and energy conversion efficiency of the resulting printed devices [149].

A further significant obstacle lies in the innovation of conductive and stretchable inks for fabricating electrodes. Conventional conductive materials, including silver or copper inks, often encounter issues related to oxidation, limited flexibility, and high processing costs. New types of materials, such as carbon-based nanomaterials [like graphene and carbon nanotubes] and inks made from liquid metals [gallium–indium alloys], present exciting opportunities; however, ensuring their stability, printability, and compatibility with polymer matrices remains an ongoing challenge [189]. Recent advancements have explored hybrid material formulations that incorporate multiple functional fillers to achieve a balance between electrical conductivity, mechanical strength, and environmental stability. Nevertheless, optimizing these inks requires extensive refinement, increasing the complexity associated with the development of 3D-printed NGs [176].

### 10.2. Scalability and Manufacturing Limitations

While three-dimensional [3D] printing offers a customizable and expeditious prototyping methodology for nanogenerators, the escalation of this technology to facilitate mass production poses a considerable challenge. The preponderance of scholarly inquiry regarding 3D-printed nanogenerators [NGs] has concentrated on devices of diminutive scales, frequently within laboratory environments; however, the transition from proof-of-concept prototypes to commercial-scale production is obstructed by numerous factors [190].

A principal constraint relates to printing velocity. Advanced 3D printing methods, such as Digital Light Processing [DLP] and stereolithography [SLA], offer exceptional feature clarity but are characterized by long processing times and limitations in output volumes [167]. In contrast, extrusion-based techniques like fused deposition modeling [FDM] and direct ink writing [DIW] enable multi-material printing but face challenges associated with layer adhesion, low resolution, and slow deposition speeds [168].

Furthermore, the incorporation of multifunctional materials within a single 3D-printed nanogenerator continues to pose challenges. Energy-harvesting devices often require the use of multiple layers composed of diverse materials—triboelectric layers, piezoelectric fillers, conductive electrodes, and encapsulation coatings. Achieving seamless integration while maintaining mechanical and electrical integrity is complicated by incompatibilities in curing temperatures, interfacial bonding, and material shrinkage [169]. Another significant concern is the reproducibility and consistency of printed nanogenerators. Small changes in parameters related to printing, such as the thickness of layers, the temperature during printing, and the curing time, may result in notable discrepancies in device performance [196]. This variability makes it difficult to ensure that large-scale manufacturing will produce uniform, high-efficiency devices, thus highlighting the need for standardized fabrication protocols and process control mechanisms [175].

### 10.3. Long-Term Stability and Degradation Issues

The enduring reliability of three-dimensional-printed nanogenerators poses a significant concern, especially regarding applications in wearable electronics, biomedical sensors, and environmental energy harvesting. The ongoing mechanical stresses and environmental conditions that these devices frequently encounter often lead to performance deterioration over extended periods [176].

A principal mechanism underlying degradation in triboelectric nanogenerators [TENGs] is the reduction in the surface charge density caused by material wear and contamination. As triboelectric layers undergo continuous contact–separation cycles, changes in their surface morphology take place, thereby reducing their ability to maintain charge accumulation. This issue is further exacerbated in flexible and stretchable nanogenerators, where bending and stretching lead to microcracks, delamination, and mechanical fatigue [174]. In response to this challenge, researchers are exploring self-healing polymers and advanced surface engineering techniques to enhance the durability of printed triboelectric layers [193].

Similarly, piezoelectric nanogenerators [PENGs] are vulnerable to polarization decay, where the alignment of dipoles in piezoelectric materials progressively deteriorates, leading to a reduced energy conversion efficiency. This phenomenon is particularly pronounced in poly [vinylidene fluoride] [PVDF]-based PENGs, as the exposure to moisture, temperature fluctuations, and mechanical stress accelerates the depolarization process [169]. Proposed encapsulation strategies that utilize hydrophobic coatings or multilayer architectures aim to improve longevity; however, these methods add complexity and costs to the manufacturing process [196].

Beyond material degradation, electrical stability presents a significant concern. Conductive electrodes in printed nanogenerators, particularly those using metallic or carbon-based inks, may suffer from oxidation, delamination, or percolation loss, which can adversely affect the overall performance [175]. The development of self-repairing and corrosion-resistant conductive materials is a critical research avenue aimed at enhancing the robustness of three-dimensional-printed nanogenerators for sustained use [191].

### 10.4. Environmental Impact and Sustainability Consideration

As the use of three-dimensional printing technology grows more common in energy-harvesting applications, concerns about material sustainability and environmental effects are also on the rise. The wide variety of polymers frequently used in 3D printing, such as acrylates, epoxies, and thermoplastics, are characterized by their resistance to biodegradation and their petroleum-based origins, leading to significant challenges related to the increase in electronic waste [165]. Furthermore, certain high-performance materials, like perovskites and metal oxides, may pose toxicity risks if not properly subjected to recycling processes [191].

The push for establishing biodegradable and eco-friendly nanogenerators [NGs] has led to the exploration of inks derived from natural polymers such as cellulose, silk fibroin, and chitosan, which show promise as alternatives to traditional synthetic materials [176]. However, these materials often exhibit an inadequate mechanical durability and electrical performance, necessitating compromises between sustainability and functionality [174]. Moreover, the energy consumption linked to certain 3D printing techniques, particularly those utilizing laser technology, raises concerns about the overall carbon footprint associated with the production of 3D-printed nanogenerators [192].

### 10.5. Interface Engineering Challenges

The field of interface engineering is vital yet challenging for enhancing high-performance three-dimensional (3D) printed nanogenerators, primarily due to the complexities involved in establishing a strong mechanical, electrical, and chemical compatibility among various materials. A paramount challenge encountered is interfacial delamination, which arises from an inadequate adhesion between sequentially printed layers or dissimilar material domains, thereby jeopardizing the integrity of the device under mechanical deformation or cyclic loading [161]. In numerous instances, the incorporation of hybrid or composite materials, including metal oxides, polymers, and carbon-based nanostructures, introduces variations in thermal expansion, surface energy, and the mechanical modulus, culminating in stress concentration and the potential for interface failure [162]. An additional persistent concern relates to inadequate control over the microstructural alignment and phase distribution at the interface, particularly in systems where the piezoelectric or triboelectric performance depends on oriented domains or phase purity [163]. Furthermore, the presence of voids or discontinuities at the interface, often created during the layer-by-layer printing process, can impair charge transport pathways and diminish the overall energy conversion efficiency [159]. Moreover, thermal and chemical incompatibilities between adjacent materials during the printing or post-processing phases may further exacerbate interfacial instability, particularly within systems subjected to repeated thermal cycling or environmental fluctuations [164]. Addressing these challenges necessitates the implementation of advanced strategies such as atomic interface engineering, functional interlayers, and the use of interpenetrating polymer networks to ensure robust bonding, seamless energy transfer, and the long-term reliability of 3D-printed nanogenerators [161].

## 11. Application of 3D-Printed Nanogenerators as Energy-Harvesting Devices

**Figure 22:**
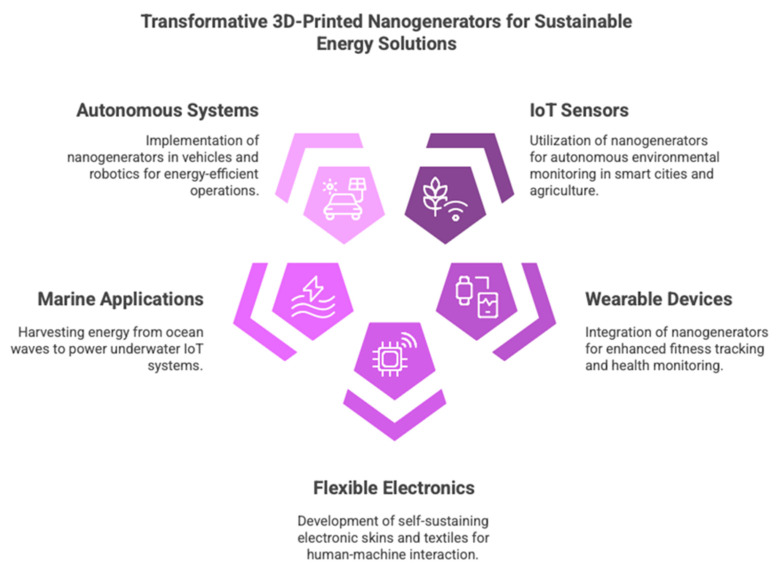
Application of 3D-printed nanogenerators.

3D-printed nanogenerators have revolutionized flexible energy harvesting by enabling self-powered systems that can efficiently convert mechanical energy into electrical energy. These devices, based on triboelectric and piezoelectric mechanisms, are widely used in wearable electronics, soft robotics, and biomedical applications due to their lightweight, stretchable, and adaptable structures. An overview of the application of 3D-printed nanogenerators in energy harvesting is shown in Figure 22.

### 11.1. IoT Sensors for Smart Cities and Agriculture

The incorporation of three-dimensional-printed nanogenerators within Internet of Things [IoT] sensors has fundamentally transformed autonomous power generation for sensing systems, thereby presenting eco-friendly energy alternatives for both smart city infrastructures and agricultural practices. These nanogenerators proficiently capture mechanical energy from ambient environmental elements, facilitating an uninterrupted functionality devoid of external energy sources [215]. Within smart city frameworks, autonomous sensors that harness triboelectric nanogenerators [TENGs] significantly enhance the immediate analysis of atmospheric contaminants, traffic density, and the condition of infrastructures [216]. The utilization of these energy-harvesting devices substantially enhances both the operational efficiency and durability of smart urban infrastructures by obviating the necessity for regular battery replacements, rendering them exceptionally suitable for extensive applications [217]. In addition, innovations in bio-inspired self-sustaining sensors have augmented the precision of environmental change detection, thereby yielding reliable data pertinent to urban development and sustainability strategies [143].

In the agricultural domain, hybrid electromagnetic–triboelectric nanogenerators [EM-TENGs] have made the detailed observation of soil moisture levels, crop health, and pest threats possible, consequently refining irrigation and resource allocation strategies [218]. The advancement of triboelectric materials derived from waste has further bolstered the sustainability of these systems, mitigating electronic waste while ensuring an effective energy transformation [219]. Also, the implementation of self-sufficient wireless sensors in precision farming has enabled the live surveillance of ecological variables, ultimately enhancing the yield in both agriculture and sustainable practices [220].

### 11.2. Wearable Devices: Fitness Trackers and Smartwatches

Wearable electronics, encompassing fitness trackers and smartwatches, are enhanced by the incorporation of 3D-printed nanogenerators that harness thermal energy and kinetic motion for the purpose of power generation. These advanced devices utilize piezoelectric and triboelectric principles to transmute biomechanical energy into electrical energy, thereby facilitating prolonged battery longevity and improved portability [221]. The amalgamation of stretchable and flexible materials within nanogenerators significantly augments their capacity to adapt to human locomotion, thereby optimizing user comfort and operational efficiency [220].

Recent empirical investigations have substantiated the viability of 3D-printed piezoelectric microdevices for biosensor applications, enabling continuous health monitoring without the need for frequent recharging [222]. Furthermore, smart textiles integrated with triboelectric nanogenerators [TENGs] have shown considerable promise for autonomous physiological monitoring, making them suitable for use in athletic performance, healthcare, and rehabilitation contexts [223]. Research focused on multi-purpose wearable devices has highlighted the potential for energy extraction from the human body to enable real-time activity observation, thereby enhancing the effectiveness of fitness tracking systems [224]. The use of additively manufactured nano-mechanical energy-harvesting systems has expanded the possibilities for energy collection in wearable technology, improving their reliability and durability [225].

### 11.3. Flexible and Stretchable Electronics

3D-printed nanogenerators are essential for the development of flexible and stretchable electronic systems, enabling the creation of self-sustaining electronic skins and intelligent textiles. These nanogenerators, integrated into wearable technology, effectively harness mechanical energy produced from physical movements, ensuring reliable energy sources for electronic skin applications and various biomedical devices [226]. The integration of stretchable smart fibers and textiles has expanded the range of nanogenerator applications within human–machine interaction frameworks, where seamless energy harvesting significantly enhances both functionality and longevity [85].

Recent innovations in shape-adaptable multifunctional flexible nanogenerators, employing coaxial direct ink writing 3D printing techniques, have markedly elevated the efficiency of energy-harvesting mechanisms [227]. Furthermore, 3D-printed intelligent gloves featuring pyramidal MXene/Ecoflex composite-based toroidal triboelectric nanogenerators [TENGs] have exhibited their relevance in wearable human–machine interaction systems, thereby facilitating improved gesture recognition and instantaneous data transmission. These breakthroughs possess the capacity to fundamentally transform the domains of assistive technology and interactive electronics, rendering devices increasingly responsive and adaptable to the requirements of users [228].

### 11.4. Marine and Underwater Applications

The potential applications of 3D-printed nanogenerators encompass marine and underwater domains, wherein they assume a pivotal function in the harvesting of energy from oceanic wave motions. Flexible triboelectric nanogenerators [TENGs] resembling seaweed and origami-configured devices have been meticulously engineered to capture energy from ongoing aquatic movements, thereby powering marine Internet of Things [IoT] systems [227,229]. The capability to harness energy from marine environments has yielded a sustainable energy source for underwater sensing and monitoring initiatives, thereby obviating the necessity for conventional battery systems [230].

Recent advancements in guided-liquid-based hybrid TENGs have exhibited superior performances in omnidirectional wave energy harvesting, thereby guaranteeing a sustainable power supply for underwater sensors and communication infrastructures [231]. The incorporation of 3D-printed double roller-based TENGs has further augmented the efficacy of blue energy harvesting, thereby facilitating progress in oceanic monitoring systems [232]. These energy-harvesting devices are anticipated to assume a crucial role in powering autonomous underwater vehicles and conducting extensive oceanographic studies, thereby contributing significantly to climate change investigations and marine conservation endeavors [225].

### 11.5. Autonomous Vehicles and Robotics

The incorporation of three-dimensional-printed nanogenerators within autonomous vehicles and robotic systems has facilitated the emergence of self-sustaining energy solutions conducive to intelligent transportation and automation. Bearing-structured triboelectric nanogenerators [TENGs] facilitate real-time vehicular monitoring, thereby enhancing safety measures and enabling predictive maintenance [228]. Implementing polymeric multilayered hybrid nanogenerators has demonstrated significant potential in self-powered vibration sensors tailored for automotive Internet of Things [IoT] applications, ensuring proficient energy harvesting from vehicular motion [233]. Moreover, 3D-printed attachment mechanisms have been investigated to optimize energy harvesting from quotidian kinetic interactions, rendering them appropriate for interactive wearable technologies [234].

In the realm of robotics, flexible nanogenerators significantly augment motion sensing capabilities and energy autonomy, thereby diminishing the reliance on external power sources [235]. The advancement of self-powered robotic systems that utilize TENGs has facilitated seamless energy generation from kinetic interactions, thus enabling prolonged operational capabilities [236]. Furthermore, contemporary research has concentrated on the integration of nanogenerators into intelligent robotic systems, enhancing their operational efficiency in industrial automation, human–robot interactions, and environmental sensing [237]. These technological innovations represent a substantial progression toward energy-efficient and sustainable autonomous systems, thereby laying the groundwork for the forthcoming generation of smart transportation and robotics [238].

In summary, 3D-printed nanogenerators have emerged as transformative devices for energy harvesting, facilitating sustainable power solutions across a diverse array of applications. Their incorporation within IoT sensors, wearables, marine technologies, and autonomous systems signifies a paradigm shift in self-powered electronic systems, paving the way for future advancements in energy-efficient technologies. The ongoing evolution of additive manufacturing methodologies and material innovations will further augment the capabilities of these devices, ultimately shaping the future landscape of self-powered electronic systems.

## 12. Conclusions

The launch of 3D-printed nanocomposites has initiated a significant shift in the development of nanogenerators, delivering marked benefits compared to traditionally crafted nanocomposites. Through the process of additive manufacturing, 3D printing facilitates meticulous control over the material composition, structural architecture, and functional attributes, culminating in enhanced energy harvesting efficiencies. The layer-by-layer fabrication methodology permits the realization of complex microstructural designs, augmented mechanical properties, and the superior integration of functional materials, thereby rendering 3D-printed nanocomposites exceptionally suitable for applications within the domain of nanogenerators.

The essential advantage of nanocomposites produced via 3D printing is their potential for maximizing the energy harvesting effectiveness by managing nanoparticle dispersion, customizing piezoelectric and triboelectric attributes, and integrating electrodes seamlessly. These attributes contribute to a heightened efficiency and performance consistency, which are imperative for practical implementations in self-powered devices, biomedical sensors, and wearable electronic systems. Conversely, conventionally fabricated nanocomposites are predicated upon traditional processing methodologies, such as melt blending, electrospinning, and casting, which frequently yield an uneven material distribution, elevated material wastage, and constrained structural flexibility. These limitations obfuscate their scalability and economic viability in large-scale energy-harvesting applications.

Moreover, the capabilities for customization embedded in 3D printing promote the assembly of hybrid nanogenerators that merge several energy conversion mechanisms, which include piezoelectric, triboelectric, and pyroelectric influences. This function not only raises the complete energy output but also broadens the possibilities for applications, featuring next-generation intelligent textiles, IoT-based products, and biomedical sensors designed for implantation. The capacity to produce flexible and wearable nanogenerators further underscores the promise of 3D-printed nanocomposites in the context of contemporary technological advancements.

Notwithstanding the notable advancements in 3D-printed nanogenerators, numerous challenges persist that necessitate resolution. Among the foremost concerns is environmental stability, as nanocomposites may exhibit susceptibility to humidity, temperature variations, and mechanical degradation. Researchers are actively investigating surface modifications, protective coatings, and advanced polymer matrices to enhance the durability and longevity of these materials. Furthermore, while additive manufacturing presents the advantage of diminished material wastage and a more sustainable fabrication paradigm, the prohibitive costs associated with specialized 3D printing equipment and materials may pose significant initial obstacles to widespread adoption. Addressing these constraints will be pivotal for large-scale commercialization and industrial assimilation.

Future investigations should concentrate on further optimizing material formulations, bolstering the mechanical robustness of printed nanogenerators, and advancing multi-material printing methodologies. Also, the design of environmentally sound and biodegradable nanocomposite compounds could notably augment the sustainability aspect of nanogenerator advancements. Integrating AI and machine learning into the 3D printing ecosystem could enable immediate adjustments to printing settings, thus improving the functionality and consistency of nanogenerators.

In the end, 3D-printed nanocomposites showcase an innovative stride in nanogenerator technology, ensuring a customizable, efficient, and scalable platform for energy-harvesting uses. As research within this domain continues to progress, it is anticipated that 3D printing will catalyze transformative developments in self-powered devices, unveiling new avenues for renewable energy solutions, next-generation electronics, and advanced biomedical applications. By overcoming prevailing difficulties and seizing opportunities in emerging technology, 3D-printed nanogenerators promise to make meaningful strides toward sustainable and autonomous energy infrastructures.

## Figures and Tables

**Table 1:** d33 Constants of various piezoelectric materials.

Material	Fabrication Method	d33 [pC/N]	Reference
PVDF [Polyvinylidene Fluoride]	3D Printing [FDM]	~5–15	[87]
PVDF-TrFE	Inkjet Printing	~20–35	[88]
BaTiO_3_ [Barium Titanate] Composite	SLA 3D Printing	~100–120	[89]
PZT [Lead Zirconate Titanate] Composite	Direct Ink Writing	~250–300	[90]
CBN -Based Ceramic	Traditional	~10–20	[85]
PLLA nanofiber	3D Printing	~20–30	[86]

**Table 2:** Comparative summary of key 3D-printable nanocomposite materials for nanogenerators.

Material	Strengths	Weakness	Reference
PVDF/P(VDF-TrFE)	High piezoelectricity; flexible; printable; good mechanical properties	Lower output compared to ceramics; requires poling for optimal performance	[94]
Ecoflex	Extremely stretchable; highly biocompatible; soft	Low dielectric constant; lower triboelectric output compared to rigid materials	[124]
BaTiO_3_ (Barium Titanate)	High piezoelectric constant; excellent ferroelectric properties	Brittle; difficult to print standalone without polymer matrix	[129]
ZnO Nanowires	Good piezoelectricity; compatible with flexible substrates	Mechanical degradation under long-term cycling; lower thermal stability	[135]
Carbon Nanotubes (CNTs)	High electrical conductivity; mechanical strength; flexible	Aggregation issues; uniform dispersion is challenging	[109]
Graphene/rGO	Exceptional conductivity; high mechanical flexibility	Requires functionalization for good matrix dispersion	[112]
MXenes	High conductivity; large surface area; hydrophilic	Susceptible to oxidation; stability concerns in humid environments	[138]
TPU (Thermoplastic Polyurethane)	High elasticity; wear resistance; good triboelectric properties	Moderate energy conversion efficiency; softer than some alternatives	[119]
PDMS	Excellent elasticity and environmental stability; biocompatible	Low intrinsic conductivity; requires fillers for enhanced properties	[110]
Nanocellulose	Renewable; biodegradable; excellent mechanical reinforcement	Poor inherent conductivity; requires blending with active fillers	[152]
Silk Fibroin	Biodegradable; biocompatible; mechanically strong	Lower piezoelectric output; needs doping or composites for energy harvesting	[154]
Chitosan	Biodegradable; antibacterial; good for biomedical devices	Limited electrical performance; requires functionalization for TENG/PENG applications	[155]

**Table 3:** Recent case studies on 3D-printed nanocomposite nanogenerators.

Sl. No.	Material System	3D Printing Technique	Nanogenerator Type	Output Performance	Environmental Conditions	Application	References
1	BaTiO_3_-PVDF composite	Direct Ink Writing [DIW]	PENG	120 V, 1.8 μA/cm^2^	Room temperature, in some cases performed under low humidity	Wearable sensors	[80]
2	ZnO-PDMS nanocomposite	Extrusion-based 3D Printing	PENG	6 V, 0.5 μA	Ambient temperature and relative humidity of 40–60%	Soft robotics	[81]
3	MXene-PVDF composite	Inkjet printing	Hybrid PENG-TENG	30 V, 2.5 μA	Room temperature, relative humidity <40%	Human motion harvesting	[146]
4	CNT-PDMS composite	SLA 3D printing	TENG	80 V, 12 μA	Room temperature with minimal ambient interference	Wearable electronics	[78]
5	AgNW-TPU composite	DIW	TENG	50 V, 8 μA	~25 °C, 40–60% relative humidity	Pressure sensors	[79]
6	BaTiO_3_-Ecoflex nanocomposite	FDM	PENG	18 V, 1.2 μA	Ambient temperature, body motion simulation	Biomechanical sensors	[141]
7	ZnO-MXene-PVDF composite	DIW	Hybrid	70 V, 3.1 μA	30–90% relative humidity, stable under humidity variation	Self-powered health monitoring	[114]
8	CNT-TPU composite	Inkjet printing	TENG	40 V, 10 μA	Ambient lab conditions	Wearable stretchable devices	[119]
9	Self-healing hydrogel nanocomposite	DIW	TENG	35 V, 1.5 μA	Tested from −20 °C to room temperature	Smart textiles	[125]
10	BaTiO_3_-PDMS composite	Robocasting	PENG	100 V, 2.8 μA/cm^2^	−10 °C to 60 °C, stable output	Structural monitoring	[31]
11	Conductive hydrogel-TPU	DIW	TENG	22 V, 0.8 μA	Physiological conditions	Biomedical sensors	[126]
12	BaTiO_3_-rGO-PVDF composite	Inkjet	PENG	90 V, 3.5 μA	Room temperature, protected against humidity	Wearable electronics	[116]
13	Chitosan-PVDF nanocomposite	DIW	PENG	14 V, 0.9 μA	37 °C (physiological)	Implantable devices	[155]
14	BaTiO_3_/PU composite	FDM	PENG	45 V, 1.0 μA	Mild humidity	Self-powered IoT devices	[127]
15	MXene-PDMS composite	Inkjet printing	TENG	60 V, 7 μA	Ambient temperature	Flexible electronics	[109]
16	Silk fibroin-PVDF	Extrusion printing	TENG	20 V, 0.5 μA	Biocompatible, 37 °C	Biodegradable energy harvesters	[157]
17	Waste plastic-graphene composite	FDM	TENG	25 V, 1 μA	Room temperature	Sustainable TENGs	[34]
18	BaTiO_3_-MXene-PVDF composite	DIW	Hybrid	85 V, 4 μA	Moderate humidity	Multi-source energy harvesting	[130]
19	CNT-Ecoflex composite	SLA	TENG	38 V, 0.9 μA	50–60% RH, room temperature	Flexible wearable systems	[23]
20	P[VDF-TrFE]-aerogel composite	DIW	PENG	65 V, 2.0 μA	Room temperature, under minimal temperature fluctuations	Motion sensors	[37]

**Table 4:** Performance comparison of 3D-printed nanocomposites.

Nanogenerator Type	Material System	3D Printing Technique	Output Voltage [V]	Output Current [μA]	Power Density	Application	References
PENG	BaTiO_3_-PVDF composite [75]	Direct Ink Writing [DIW]	120	1.8	3.5 mW/cm^2^	Wearable sensors	[80]
PENG	ZnO-PDMS composite [76]	Extrusion-Based 3D Printing	6	0.5	0.15 mW/cm^2^	Soft robotics	[81]
Hybrid [PENG-TENG]	MXene-PVDF composite [77]	Inkjet Printing	30	2.5	0.7 mW/cm^2^	Human motion harvesting	[82]
TENG	CNT-PDMS composite [73]	SLA 3D Printing	80	12	2.5 mW/cm^2^	Wearable electronics	[78]
TENG	CNT-TPU composite [103]	Inkjet Printing	40	10	1.2 mW/cm^2^	Stretchable wearables	[119]
Hybrid	ZnO-MXene-PVDF composite [57]	DIW	70	3.1	2.8 mW/cm^2^	Self-powered health monitoring	[58]
PENG	BaTiO_3_-TPU composite [31]	Robocasting	100	2.8	3.0 mW/cm^2^	Structural monitoring	[31]
TENG	Self-healing hydrogel-TPU [109]	DIW	35	1.5	0.4 mW/cm^2^	Smart textiles	[125]
PENG	BaTiO_3_-rGO-PVDF composite [100]	Inkjet Printing	90	3.5	3.2 mW/cm^2^	Wearable electronics	[116]
TENG	MXene-PDMS composite [93]	Inkjet Printing	60	7	1.8 mW/cm^2^	Flexible electronics	[109]
PENG	Chitosan-PVDF composite [137]	DIW	14	0.9	0.18 mW/cm^2^	Implantable devices	[155]
TENG	CNT-Ecoflex composite [23]	SLA	38	0.9	0.95 mW/cm^2^	Flexible wearable systems	[23]

**Table 5:** Advantages and challenges of 3D-printed nanocomposites.

Advantages	Challenges
Customization and complex geometries for optimized designs	Limited material printability and nanoparticle dispersion issues
Versatility in material selection for enhanced properties	Brittle nature of some functional materials
Cost-effective and scalable production	Mechanical degradation under prolonged use
Integration of conductive electrodes within the structure	Environmental factors affecting long-term stability
Enhanced flexibility and stretchability for wearable applications	Challenges in achieving high energy conversion efficiency
Sustainable fabrication with reduced material wastage	Need for multifunctional hybrid designs to enhance performance

**Table 6:** Comparison of properties of 3D-printed nanocomposites and nanocomposites made using conventional methods.

Property	3D-Printed Nanocomposites	Nanocomposites
Fabrication Method	Additive manufacturing, layer-by-layer deposition [165]	Traditional methods like melt blending, electrospinning, and casting [75]
Material Integration	Multi-material printing allows for functionalized nanocomposites [176]	Requires multiple processing steps for material integration [76]
Structural Complexity	Can produce highly complex and customized designs [166]	Limited by conventional fabrication constraints [76]
Mechanical Properties	Improved due to precise microstructural control [167]	Dependent on fabrication technique, may have inconsistencies [78]
Energy Harvesting Efficiency	Enhanced due to optimized micro-architectures [174]	Relies on bulk material properties and limited design optimization [79]
Piezoelectric Performance	Higher due to well-controlled nanoparticle dispersion [167]	Affected by nanoparticle agglomeration and uneven dispersion [113]
Triboelectric Performance	Can be enhanced by precise material layering [94]	Less controlled layering results in variations [111]
Scalability	Scalable using advanced printing methods like multi-jet fusion [172]	Requires batch processing, increasing material waste and cost [160]
Flexibility	High, especially for wearable applications [168]	Dependent on material properties, usually requires additional processing [138]
Electrode Integration	Can be printed directly into the structure [174]	Requires manual assembly and additional processing [160]
Environmental Stability	Susceptible to humidity and temperature changes but can be improved with coatings [80]	Material degradation due to exposure to environmental factors [137]
Sustainability	Reduced material waste, more eco-friendly fabrication [130]	Higher material consumption and waste generation [139]
Cost Efficiency	Lower production costs due to minimal waste and fewer fabrication steps [174]	Higher costs due to multi-step processing and material loss [188]
Performance Consistency	High due to precise control over material composition and architecture [185]	Can vary due to non-uniform fabrication methods [140]
Hybrid Nanogenerators	Easily integrates multiple energy-harvesting mechanisms [93]	Requires complex assembly for hybrid designs [151]
Wearable Applications	Ideal due to conformal and flexible design capabilities [187]	Limited by material rigidity and manufacturing constraints [114]
Future Potential	Advancements in additive manufacturing can further enhance properties [17]	Continuous improvements in materials and fabrication techniques required [109]

**Table 7:** Comparative analysis of additive manufacturing techniques.

Method	Material Compatibility	Cost	Resolution	Key Advantages	Limitations
DIW	Broad (inks, nanocomposites, polymers)	Low	~100 μm	Room temperature processing, good for soft materials	Lower resolution; ink formulation is critical
FDM	Thermoplastics (e.g., TPU, PLA, ABS)	Very Low	~200–300 μm	Simple, low-cost, rapid prototyping	Poor resolution; high temp required
SLA	Photocurable resins	Medium	~50 μm	High resolution, smooth surfaces	Limited resin choices; brittle materials
DLP	Photopolymers, composites with nanoparticles	Medium	~25–50 μm	Faster than SLA, finer details	Resin must be photoactive; post-processing often needed

**Table 8:** Comparison of 3D-printed vs. conventional nanogenerators.

Parameter	3D-Printed Nanogenerators	Conventional Nanogenerators	Reference[s]
Fabrication Method	Additive manufacturing [e.g., DLP, SLA, FDM] enables rapid prototyping and complex geometries	Conventional methods include photolithography, chemical vapor deposition, and electrospinning, which are labor-intensive and require multiple steps	[39,168,174]
Material Selection	Wide range of materials, including biodegradable polymers, nanocomposites, and conductive inks	Primarily relies on pre-synthesized nanomaterials and traditional substrates, limiting flexibility	[169,176,183]
Cost Efficiency	Reduced production cost due to minimal material wastage and single-step processing	High fabrication costs due to complex etching, deposition, and assembly processes	[42,149]
Energy Output [Voltage and Power Density]	Optimized microstructures and controlled material composition enhance charge transfer, increasing efficiency	Traditional techniques achieve high power output but require post-processing for enhanced triboelectric performance	[44,45,49]
Flexibility and Wearability	Highly flexible and stretchable due to printable elastomeric and conductive materials	Limited flexibility, often requiring hybrid structures for enhanced mechanical properties	[52,147,168]
Scalability	Scalable manufacturing for mass production using automated 3D printing	Challenging to scale due to reliance on batch processes and complex alignment techniques	[167,190]
Integration with Other Systems	Easily integrates with flexible electronics, sensors, and self-powered devices	Requires additional interfacing steps for hybrid system integration	[51,54]
Mechanical Durability	Resilient under repeated mechanical stress due to the use of stretchable and self-healing materials	Prone to mechanical degradation under cyclic loading	[64,176]
Sustainability and Environmental Impact	Biodegradable and eco-friendly 3D-printed materials reduce environmental footprint	Traditional materials may generate toxic waste and are difficult to recycle	[176,187]
Application Versatility	Used in smart textiles, biomedical devices, soft robotics, and self-powered sensors	Primarily employed in energy harvesting and sensor networks, with limited adaptability to wearables	[56,58]

## Data Availability

The original contributions presented in this study are included in the article. Further inquiries can be directed to the corresponding author.
